# Multiple Charged Purine (MCP) PNA as a Simple Mode for Cellular Uptake

**DOI:** 10.1021/acspolymersau.6c00012

**Published:** 2026-04-21

**Authors:** Salam Maree, Eylon Yavin

**Affiliations:** The Institute for Drug Research, The School of Pharmacy, The Faculty of Medicine, 108406 The Hebrew University of Jerusalem, Hadassah Ein-Kerem, Jerusalem 9112102, Israel

**Keywords:** PNA, cationic purines, cellular uptake, CPP-PNA, *N*-methylation

## Abstract

Peptide nucleic acid (PNA) is a synthetic DNA analogue characterized by exceptional biostability and strong hybridization affinity toward complementary DNA and RNA. However, its inherently low membrane permeability hampers its biomedical applicability. *N*-Methylation of guanine and adenine PNA monomers produces a positively charged nucleobase, which suppresses the formation of self-duplexes while improving DNA affinity through electrostatic interactions. To overcome the cellular delivery limitations of PNA, we designed and synthesized a 16-mer, nontargeted model PNA incorporating 4, 6, or 8 positively charged purines (G^+^ and A^+^). As comparative controls, the corresponding unmodified PNA was conjugated to a short cell-penetrating peptide (CPP) containing 4, 6, or 8 d-lysine residues. All constructs were labeled with Rhodamine B to enable quantitative cellular uptake analysis. Flow cytometry and confocal microscopy in OVCAR-8 ovarian cancer cells revealed that the PNA incorporating six positively charged purines (MCP6, multiple charged purines with an overall 6 positive charges) exhibited markedly enhanced cellular internalization compared to both the other MCP-PNAs and the CPP-PNA controls. MCP-PNAs showed no noticeable signs of cell toxicity, and their binding affinities (thermal melting profiles) were comparable to CPP-PNAs. In addition, MCP-PNAs well discriminated single mismatches in RNA, similarly to CPP-PNAs. Overall, this strategy provides a simple and effective approach for generating inherently cell-permeable PNAs.

## Graphical abstract

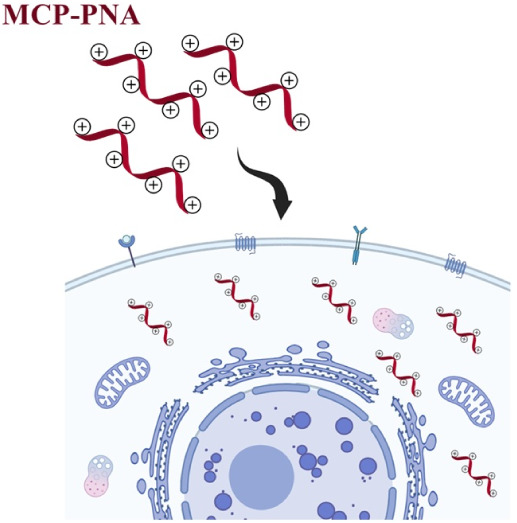

## Introduction

Synthetic oligonucleotides have become integral to a wide range of scientific disciplines, including organic chemistry, molecular biology, drug discovery, genetic diagnostics, nanotechnology, and even prebiotic chemistry. This broad utility has motivated the development of numerous nucleic acid analogues designed to overcome the intrinsic limitations of natural DNA and RNA. Among these, peptide nucleic acid (PNA) stands out as a particularly compelling DNA mimic.^1^ In PNA, the canonical sugar–phosphate backbone is replaced with a neutral, achiral pseudopeptide scaffold, yielding exceptionally strong hybridization to complementary nucleic acids, remarkable resistance to enzymatic degradation, and excellent single-nucleotide mismatch discrimination.^2–4^ These advantageous properties have firmly established PNAs as powerful tools for both therapeutic^5–13^ and diagnostic applications.^14–20^

However, unmodified PNAs exhibit several drawbacks, including poor aqueous solubility^4,21^ and a pronounced tendency to interact nonspecifically with hydrophobic surfaces—such as other PNA strands—leading to aggregation.^22,23^ Several strategies have been developed to address these limitations, including (1) conjugation to charged or hydrophilic groups, (2) encapsulation within nanoparticle-based carriers,^12,24–26^ and (3) the introduction of backbone modifications^27,28^ such as guanidinium-PNA (GPNA).^29–33^

Among the earliest approaches explored for enhancing intracellular delivery of PNAs was the use of cell-penetrating peptides (CPPs);^34,35^ a broad family of short peptides (typically 10–30 residues) capable of crossing cellular membranes at low micromolar concentrations without inducing significant membrane damage. In a previous study we employed CLIP-6, a CPP that enters cells through a nonendosomal mechanism and exhibits excellent biocompatibility and serum stability,^36^ to achieve efficient delivery of an 18-mer PNA into a glioblastoma cell line (U87).^37^ In an earlier report, Slack and coworkers conjugated an antimiR-155 PNA to the tumor-targeting peptide pHLIP providing a CPP-PNA with in vivo anticancer activity.^38^

Another approach for PNA cellular delivery is based on PNA conjugation to saccharides. For example, recent studies have shown potent antimicrobial activity of PNA-sugar conjugates.^39,40^ In addition, liver targeted delivery of PNA has been realized by conjugation to multivalent sugars (e.g., *N*-acetylgalactosamine, GalNAc).^41,42^

Nanomaterial-based delivery systems have also been extensively investigated. Massaro et al. described a halloysite nanotube (HNT)-based platform functionalized with halochromic oxazine dyes for fluorescence tracking, enabling the intracellular transport of a single-stranded PNA tetramer (PNAts).^43^ In this design, the PNA was covalently attached to the outer HNT surface, allowing for controlled, acid-responsive release. Beyond HNTs, PNA loading has been optimized into a variety of nanoparticle systems including silica-based nanocarriers,^44^ Graphene Oxide (GO) nanoparticles,^45^ and gold nanoparticles (AuNPs)^46,47^ to improve delivery efficiency and cellular uptake. Additionally, antimiR-155 PNA encapsulated in PLGA nanoparticles has been shown to inhibit miR-155 function and reduce its expression levels in B-cell lymphomas.^48^

Backbone modifications—particularly γ-modifications—have emerged as a powerful strategy for improving PNA solubility, structural preorganization, and biological performance.^28,49–52^ Mini-PEG γ-PNAs,^53^ for example, have been successfully applied to target diverse RNA molecules, including oncogenic microRNAs (oncomiRs).^54^ More recently, tetrahydrofuran-modified PNAs (“thyclotides”) were introduced as a new class of backbone-engineered oligomers in which ethylenediamine units are replaced with a cyclic THF scaffold, resulting in efficient cellular uptake across multiple cell types and potent inhibition of microRNA-21.^55^

Modification of nucleobases has likewise been explored, albeit far less frequently. 2-Aminopyridine modification of PNA nucleobases was shown to improve cellular uptake in HEK293 cells (human embryonic kidney cells) compared to unmodified PNAs.^56^ This modification also enhanced dsDNA invasion by enabling stable, sequence-specific PNA–DNA–PNA triplex formation under physiological conditions, where traditional base modifications were ineffective.^57^

Hibino et al. reported that incorporation of *N*-methylated guanine (G^+^) into PNA suppresses self-hybridization while simultaneously enhancing affinity toward complementary DNA.^58^ Building on this concept, we introduced multiple A^+^ and G^+^ bases (Table 1) into PNA sequences as a simple and straightforward strategy to generate cell-permeable multiple charged purine PNAs, termed MCP-PNAs. As controls, we synthesized the same nontargeted 16-mer PNA sequence conjugated to 4, 6, or 8 d-lysine residues to promote solubility and intracellular delivery. All PNAs were labeled with a Rhodamine B fluorescent tag to enable uptake monitoring.

**Table 1:**
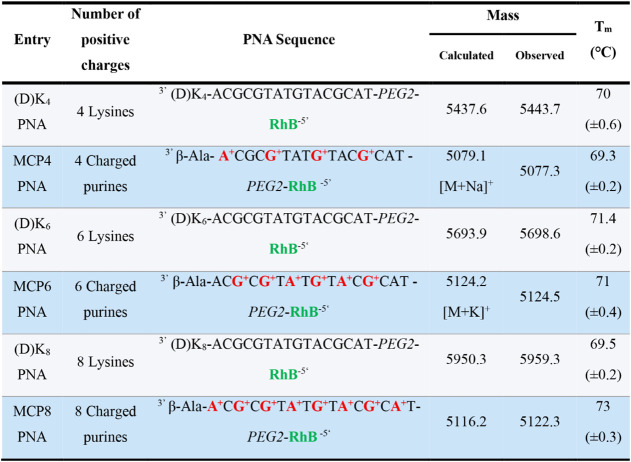
Multiple Charged Purine PNAs (MCP-PNAs) and Control CPP-PNAs^a^

a (D)­K_4_, (D)­K_6_, and (D)­K_8_ denote PNAs conjugated to 4, 6, or 8 d-lysines, respectively. Cationic purines (G^+^/A^+^) are highlighted in red, and Rhodamine B is shown in green. Melting temperatures (*T*_m_) were determined in PBS buffer (pH 7.0) following thermal annealing of PNA with the complementary RNA at a 1:1 ratio, with a final duplex concentration of 2 μM.

In this study, we demonstrate that incorporation of multiple positively charged purines into the PNA sequence promotes cellular uptake in ovarian cancer cells. All MCP-PNAs were internalized, with the construct containing six charged purines (MCP6) exhibiting the highest uptake, outperforming both other MCP-PNAs and unmodified PNAs.

## Materials and Methods

Detailed procedures for PNA synthesis and the *N*-methylation reaction of PNA A monomer are provided in the Supporting Information.

### Melting Temperatures (*T*_m_) Measurement

The thermal stability of the PNA: RNA duplexes was assessed by UV melting analysis using an Evolution One Plus UV–vis spectrophotometer. Solutions of the PNAs and their complementary synthetic RNA strand (1:1 ratio) were prepared in phosphate-buffered saline (PBS; 100 mM NaCl, 10 mM NaH_2_PO_4_, pH 7.0) at a final duplex concentration of 2 μM. Samples were initially heated from 20 °C to 90 °C at a rate of 5 °C/min, then cooled to 20 °C at 2 °C/min. Absorbance at 260 nm was continuously recorded during a subsequent heating cycle from 20 °C to 90 °C at a rate of 1 °C/min. Melting temperatures (*T*_m_) were determined from the inflection point of the melting curve and represent the average of at least two independent measurements.

### Circular Dichroism (CD) Spectroscopy

Circular dichroism (CD) spectra were recorded using a Jasco F-1100 spectropolarimeter equipped with a temperature-controlled sample holder. Measurements were conducted on both single-stranded PNA and PNA:RNA duplexes (1:1 ratio), prepared at a final PNA concentration of 15 μM in phosphate-buffered saline (PBS; 100 mM NaCl, 10 mM NaH_2_PO_4_, pH 7.0). Duplex formation was achieved by incubating the samples at 60 °C for 5 min then cooling gradually to room temperature. CD spectra were acquired at 25 °C using a 1 mm path length quartz cuvette with a total volume of 200 μL. Data were collected over the wavelength range of 200–320 nm, with each spectrum representing the average of five individual scans.

### Statistical Analysis

FACS data are presented as the mean ± SD from experiments. Three independent experiments were performed: each conducted with two or three technical replicates.

Statistical significance was determined using a one-way test, with *P* < 0.001 considered extremely significant (***), *P* < 0.01 highly significant (**), and *P* < 0.05 statistically significant (*).

### Cell Culture

OVCAR-8 and MeWo cells were cultured in EMEM and DMEM medium, respectively (Beit Haemek Biological Industries, Israel), supplemented with 10% (v/v) fetal bovine serum (FBS), 100 U/mL penicillin, 0.1 mg/mL streptomycin, and 2 mM l-glutamine. Cells were maintained at 37 °C in a humidified incubator with 5% CO_2_. Routine testing for mycoplasma contamination was performed using the MycoBlue Mycoplasma Detector Kit (Vazyme, China).

### Flow Cytometry Analysis

For flow cytometry analysis of PNAs uptake, OVCAR-8 (3.5 × 10^5^) cells and MeWo (1.95 × 10^5^) cells were seeded in 6-well plates and cultured overnight under standard conditions until reaching approximately 70–80% confluence. The culture medium was then replaced with fresh medium containing 2 μM PNAs, and cells were incubated for 5 h at 37 °C in a humidified atmosphere with 5% CO_2_. Following incubation, cells were washed thoroughly with PBS, detached using 0.25% Trypsin-EDTA (5 min at 37 °C), collected into 15 mL Falcon tubes, and centrifuged at 1200 rpm for 5 min. After discarding the supernatant, cell pellets were resuspended in 350 μL cold PBS and passed through 70 μm Falcon cell strainers. Samples were analyzed using a BD LSRFortessa flow cytometer (Core Research Facilities, The Hebrew University of Jerusalem, Israel). Gating was performed based on the fluorescence profile of untreated control cells to quantify the percentage of cells that internalized Rhodamine B labeled PNAs. Data were processed using FlowJo software (version 10.10).

### Confocal Microscopy Analysis

For live-cell imaging, OVCAR-8 (6 × 10^4^) cells were seeded into μ-slide 8-well chambers (ibidi GmbH, Gräfelfing, Germany) and cultured for 24 h at 37 °C in a 5% CO_2_ atmosphere to allow adherence and achieve 60–70% confluence. Cells were then gently washed with 1× PBS and incubated with 2 μM PNAs in complete medium for 5 h under standard conditions. Postincubation, cells were washed twice with PBS and subsequently stained with Hoechst dye (1 μg/mL) for 15 min at room temperature to visualize nuclei. Following a final wash with PBS, 300 μL of fresh PBS was added to each well to maintain cell viability during imaging. Untreated cells served as negative controls. Fluorescence imaging was performed using a Nikon AIR+ confocal microscope (Core Research Facilities, The Hebrew University of Jerusalem, Israel), and image analysis was conducted using NIS-Elements AR software (version 5.21).

For lysosomal colocalization studies, following PNA incubation, cells were washed twice with PBS and stained with LysoTracker Deep Red (75 nM) for 30 min in at 37 °C. Cells were then washed twice with PBS, stained with Hoechst dye (1 μg/mL) for 15 min at room temperature, and imaged as described above.

### Cell Viability Assay

OVCAR-8 cells were seeded in 96-well plates and cultured until approximately 60% confluency was reached. Cells were then treated with the different PNAs at three concentrations (0.1 μM, 1 μM, and 10 μM) for 48 h. Following treatment, the culture medium was removed, and the wells were gently washed with PBS. Cell fixation was carried out by adding 100 μL of 100% cold methanol to each well, followed by incubation for 20 min at room temperature. Methanol was then aspirated, and the plates were left to air-dry completely. Subsequently, cells were stained with 50 μL of 0.1% Crystal Violet solution for 30 min. Excess stain was removed, and the wells were rinsed thoroughly with PBS. Stained cells were visualized using a 4K HDMI industrial digital camera operating at 75 frames per second. All experiments were conducted in triplicate.

## Results

### Chemical Synthesis of G^+^ and A^+^

The G^+^ PNA monomer was synthesized following the procedure reported by Hibino et al.^58^ The A^+^ monomer was synthesized using the same protocol with minor modifications (see Supporting Information for experimental details). Unlike G^+^, which was obtained directly after a simple workup, A^+^ required purification by semipreparative HPLC, affording an 18% yield (Scheme 1). The final A^+^ product was characterized by ^1^H NMR, ^13^C NMR, and HRMS (Figures S29–S30).

**Scheme 1:**
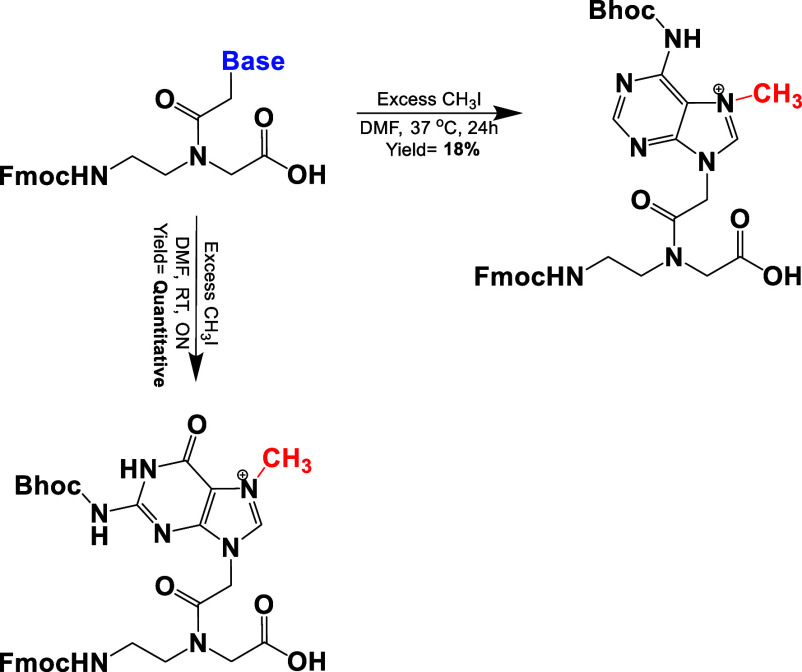
*N*-methylation of Guanine (G) and Adenine (A) PNA Monomers

### Chemical Synthesis of PNAs

To investigate whether multiple positive charges on purine bases enhance cell permeability, we designed and synthesized a 16-mer nontargeted model PNA containing 4, 6, or 8 positively charged purine modifications randomly distributed along the sequence. As controls, the same unmodified PNA sequence was conjugated to short CPPs consisting of 4, 6, or 8 d-lysines (Table 1), selected to match the number of positive charges. The choice of multiple d-lysines was based on previous reports where PNA uptake into a variety of cell lines was achieved using the simple (D)­K_4_ CPP.^16,59–63^ All PNAs were labeled with a Rhodamine B fluorophore through a PEG linker to enable monitoring cellular uptake. Synthesis was carried out on solid support (Novasyn TGA resin) using standard Fmoc-based peptide/PNA chemistry. Following cleavage from the resin, the oligomers were purified by HPLC and characterized by MALDI-TOF MS (Figures S1–S6).

### Cellular Uptake Studies in OVCAR-8 Cells

To assess cellular uptake, MCP-PNAs and CPP-PNAs were incubated with ovarian cancer cells (OVCAR-8) at 2 μM for 5 h, and uptake was analyzed by flow cytometry (Figure 1). All MCP-PNAs were internalized, although the extent of uptake varied with the number of positively charged purines. Among these, MCP6-PNA displayed the highest uptake, surpassing all other PNAs, both MCP-modified and controls, whereas MCP8-PNA showed the lowest internalization. MCP4-PNA exhibited uptake levels similar to (D)­K_6_–PNA and (D)­K_8_–PNA. Within the control group, (D)­K_4_–PNA demonstrated significantly greater uptake than the other CPP-PNA sequences.

**Figure 1:**
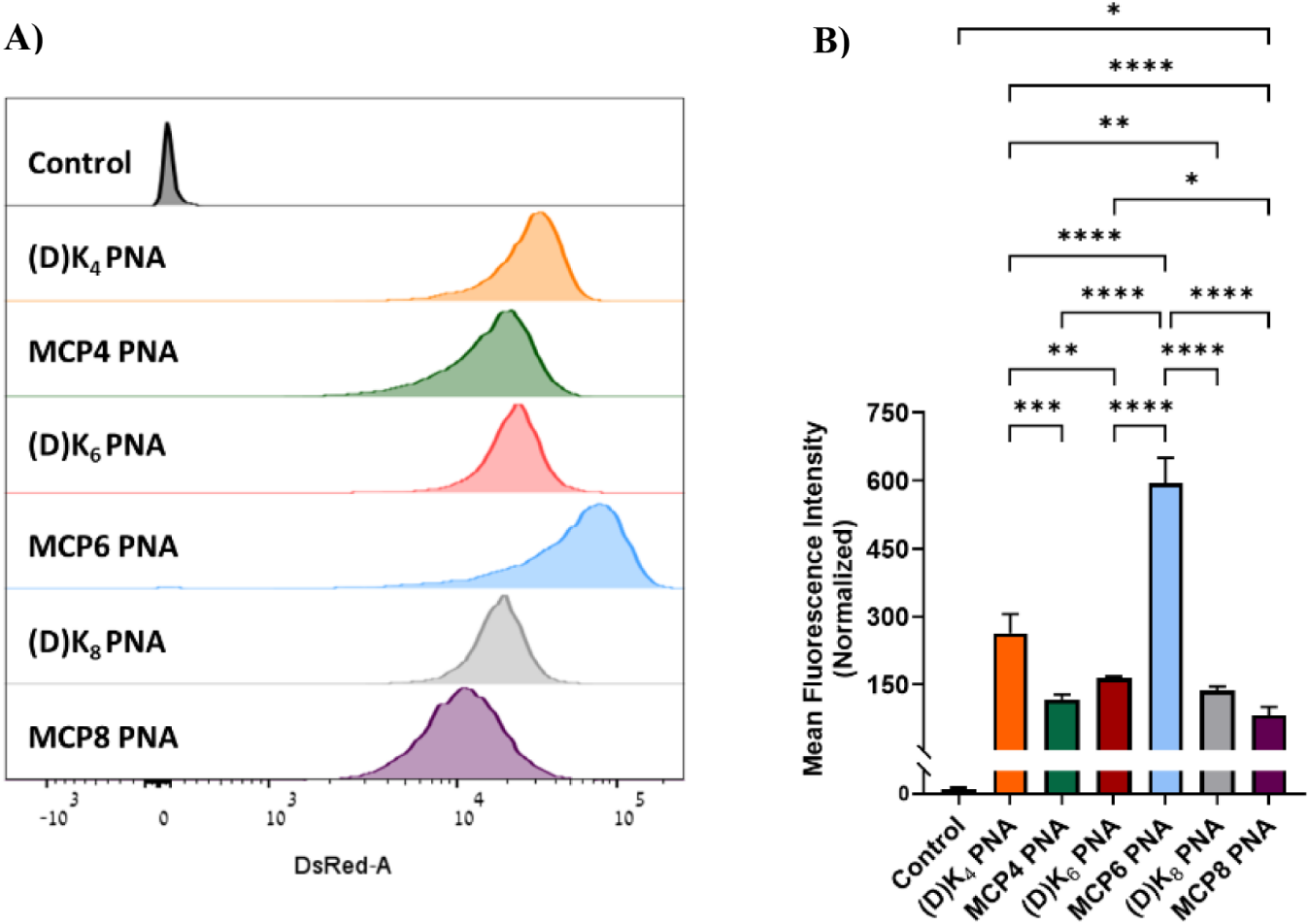
Flow cytometry analysis of OVCAR-8 cells after incubation with 2 μM MCP-PNAs and CPP-PNAs for 5 h at 37 °C. (A) Histogram of FACS analysis in OVCAR-8 cells treated with PNAs. Histogram illustrates the mean fluorescence intensity plotted in horizontal axis against the number of cell events detected in the vertical axis. (B) Mean fluorescence intensity of PNAs in OVCAR-8 cells. The Data is presented as the mean ± SD (*n* = 4). *** represents *p* ≤ 0.001, ** represents *p* ≤ 0.01 and * represents *p* ≤ 0.05 as determined by a One-way ANOVA test.

Confocal microscopy was used to visualize intracellular localization (Figure 2). OVCAR-8 cells were treated with 2 μM PNAs for 5 h and counterstained with Hoechst to label nuclei. Fluorescence signals from Rhodamine-labeled PNAs were detected for most PNAs with MCP6-PNA showing the strongest and most widespread intracellular signal, consistent with the flow cytometry results. MCP8-PNA, on the other hand, produced no detectable intracellular fluorescence, further reflecting its poor uptake efficiency.

**Figure 2:**
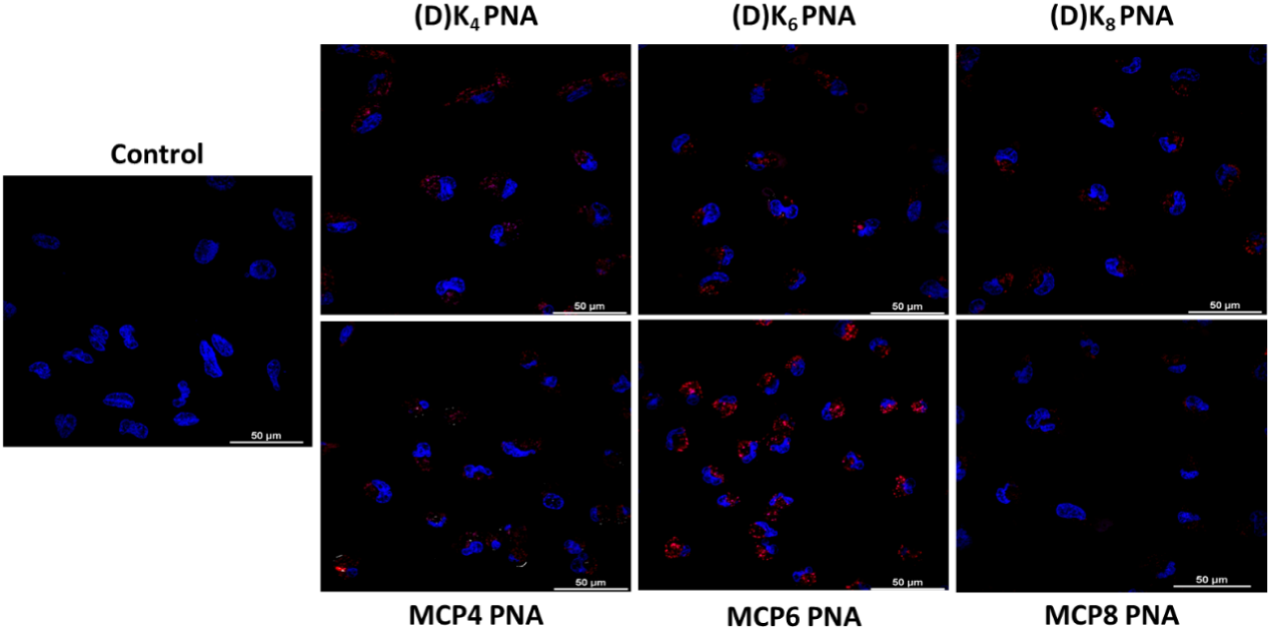
Confocal imaging of CPP-PNAs and MCP-PNAs (in red) in OVCAR-8 cells with the nucleus labeled by Hoechst marker (blue). Cells were treated with 2 μM of PNAs for 5 h at 37 °C. Untreated cells served as control. Scale bar = 50 μm.

To further elaborate cellular uptake and intracellular localization, confocal microscopy was conducted following uptake of MCP6-PNA and (D)­K_6_–PNA using the same conditions presented in Figure 2, with the addition of LysoTracker as a lysosomal marker (Figure 3, green). As presented in Figure 3, both PNAs display marked colocalization with lysosomes and are found almost exclusively in the cytoplasm (excluded from the nucleus). Nevertheless, a portion of the Rhodamine B signal (red), corresponding to PNA labeling, is detected outside the lysosomal compartment, suggesting that not all of the PNA is trapped in the lysosome.

**Figure 3:**
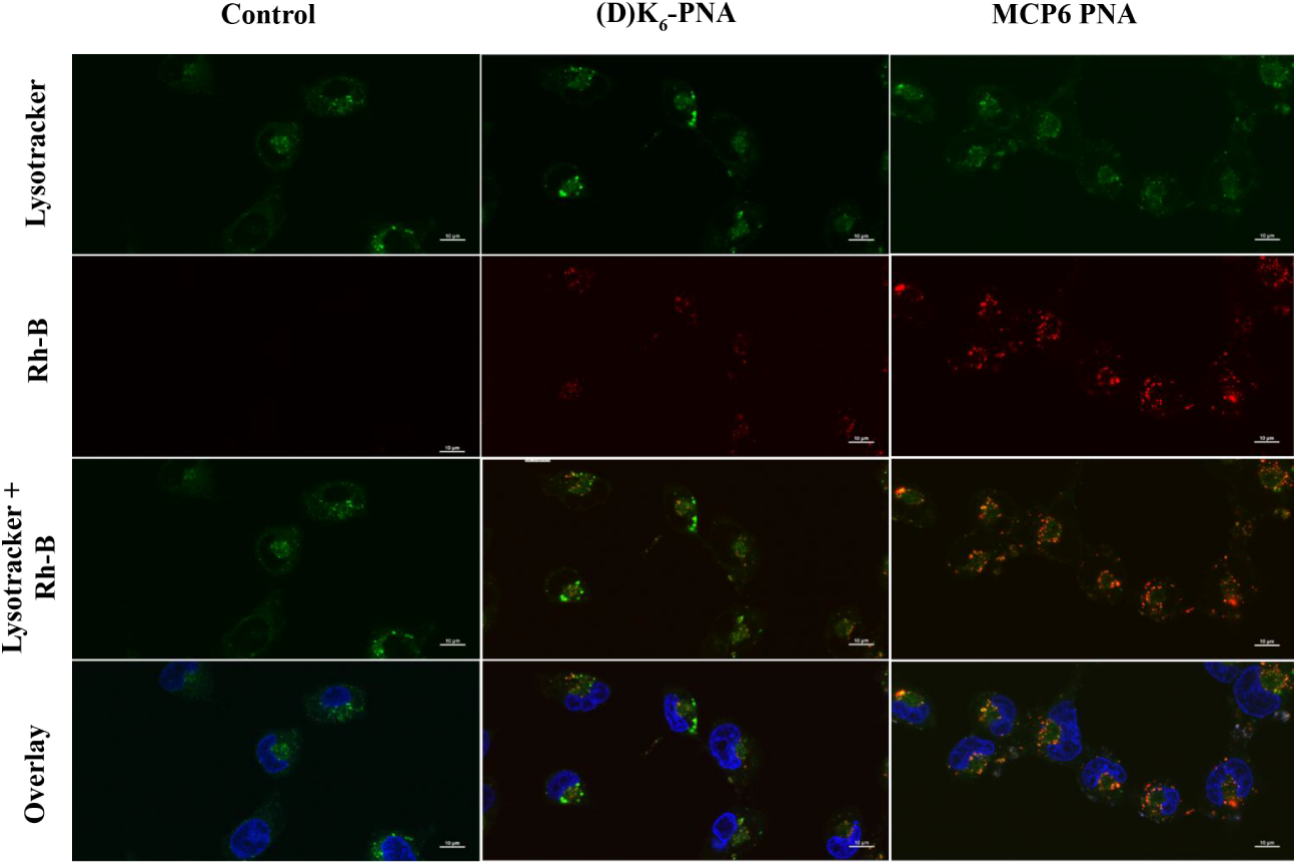
Confocal images of OVCAR-8 cells treated with (D)­K_6_–PNA and MCP6 PNA (in red), with lysosomes stained with LysoTracker Deep Red (green) and nuclei stained with Hoechst (blue). Cells were incubated with 2 μM PNA for 5 h at 37 °C. Untreated cells served as control. Scale bar = 10 μm.

To assess the potential cytotoxicity of introducing multiple positive charges into the PNA sequence, OVCAR-8 cells were treated with increasing concentrations (0.4, 2, and 10 μM) of MCP-PNAs and CPP-PNA controls for 48 h, and viability was measured using crystal violet staining (Figures S27–S28). Cell viability remained constant across all concentrations and was comparable to untreated controls, with no evidence of dose dependence or statistically significant reduction in cell viability. This outcome was observed for both MCP-PNAs and CPP-PNAs, confirming that the introduction of multiple positive charges does not induce cytotoxicity, even at high concentrations such as 10 μM. Notably, MCP6-PNA, the construct with the highest cellular uptake—was also well tolerated. Collectively, these results highlight the potential of multiple positively charged purines to enhance cellular entry, with MCP6-PNA emerging as the most effective and promising construct in OVCAR-8 cells.

### Duplex Stability and Secondary Structure Analysis

To evaluate the thermal stability of the modified PNAs, melting temperature (*T*_m_) measurements were performed with a complementary synthetic RNA. All PNAs, both MCP-modified and CPP-controls, displayed comparable melting temperatures (Table 1) with *T*_m_ values ranging from 69 to 73 °C. MCP8-PNA exhibited the highest duplex stability (*T*_m_ = 73.0 °C), ∼3.5 °C higher than its CPP counterpart corresponding to CPP control ((D)­K_8_ (69.5 °C)). These results indicate that incorporation of multiple positive charges into the PNA bases does not enhance binding affinity but, importantly, does not disrupt duplex formation or stability with the RNA target. Given its superior cellular uptake, MCP6-PNA was selected for further analysis.

Circular Dichroism (CD) spectroscopy was performed to evaluate the secondary structure and helical organization of MCP6-PNA and the control (D)­K_6_–PNA in both their single-stranded forms and after hybridization with complementary RNA (Figure S18). As expected, single-stranded PNAs exhibited minimal CD signal in the 200–300 nm range. Upon duplex formation, both MCP6-PNA:RNA and (D)­K_6_–PNA:RNA displayed spectra characteristic of antiparallel PNA:RNA heteroduplexes, with a pronounced positive peak around 265–270 nm, a minimum at ∼235 nm, and a secondary maximum between 220–225 nm. These spectral characteristics indicate a right-handed helical conformation, consistent with previously reported PNA:RNA duplexes. The overall signal amplitude and spectral profile were comparable for both constructs and aligned with the *T*_m_ data, confirming that the incorporation of six internal positive charges in MCP6-PNA does not alter the conformational dynamics or base-stacking interactions within the duplex.

To further confirm duplex formation, HPLC analysis was performed for ssRNA, PNA (MCP6-PNA/(D)­K_6_–PNA), and PNA:RNA duplexes (Figure 4). A clear decrease in ssRNA and PNA is evident for the annealed samples, and a new distinct signal appears, which is consistent with duplex formation. MALDI-TOF MS (Figures S8–S9) further indicates duplex formation for both PNAs.

**Figure 4:**
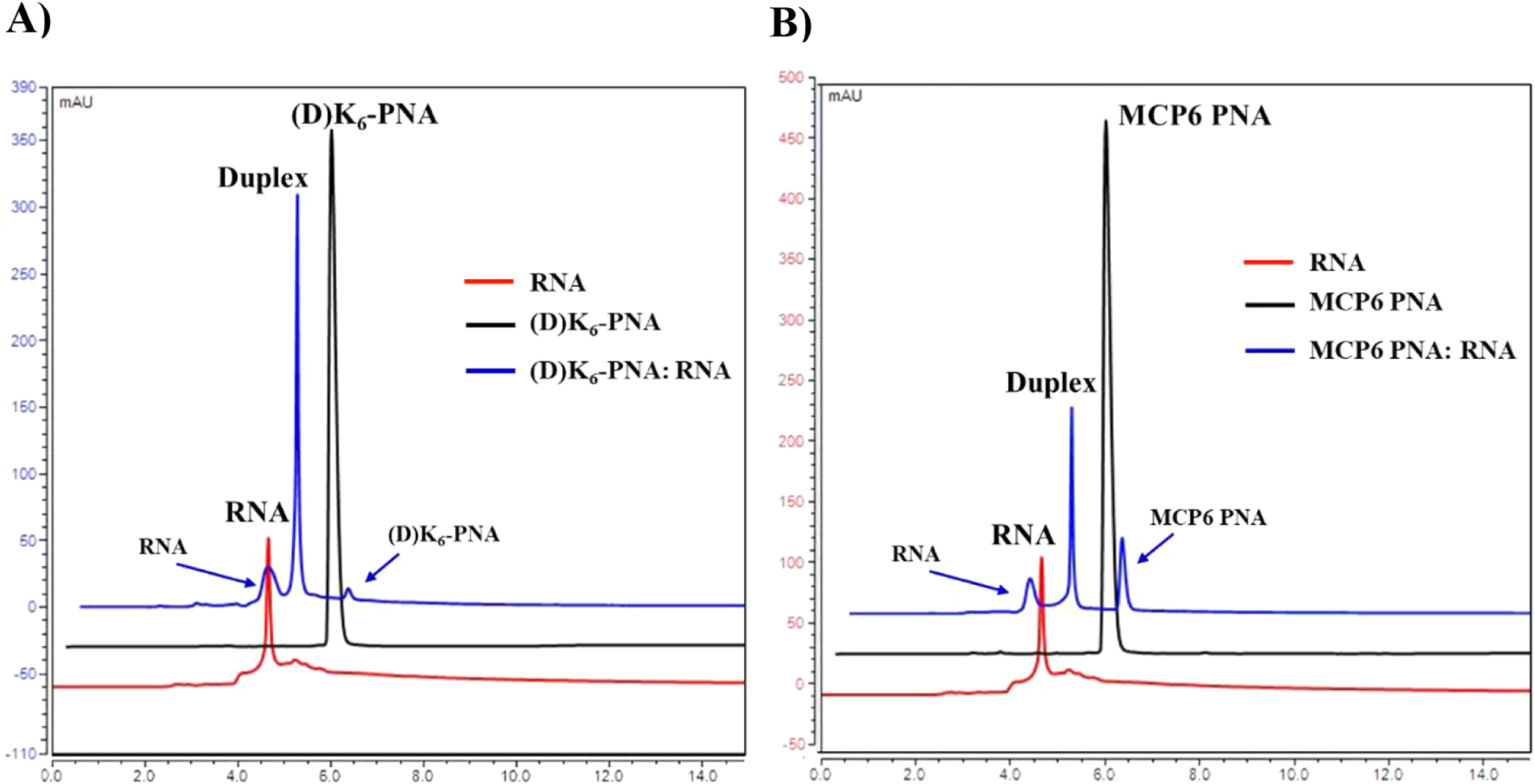
HPLC analysis of duplex formation between complementary RNA and either (D)­K_6_–PNA or MCP6-PNA, monitored by UV absorbance at 260 nm. Eluents: A (0.1% TEAA in water) and B (MeCN) were used in a linear gradient (5–25% B over 20 min) at a flow rate of 4 mL/min. (A) Chromatograms of ssRNA (red), (D)­K_6_–PNA (black), and the annealed (D)­K_6_–PNA:RNA duplex (blue). (B) Chromatograms of ssRNA (red), MCP6-PNA (black), and the corresponding MCP6-PNA:RNA duplex (blue). Duplex formation was achieved by incubating the samples in water at 60 °C for 5 min, followed by gradual cooling to room temperature. [PNA] = [RNA] = 30 μM.

The sequence specificity and mismatch discrimination capacity of MCP6-PNA were determined by measuring *T*_m_ values with a series of synthetic RNA strands containing designed mismatches, alongside the control (D)­K_6_–PNA. Single mismatches were positioned opposite the G^+^ and A^+^ residues in the PNA strand, as well as at an intervening uncharged site between them (Table 2). Additional RNA strands with reduced complementarity—containing two or three mismatches—were included to more rigorously evaluate each construct’s ability to distinguish fully matched from mismatched targets.

**Table 2:**
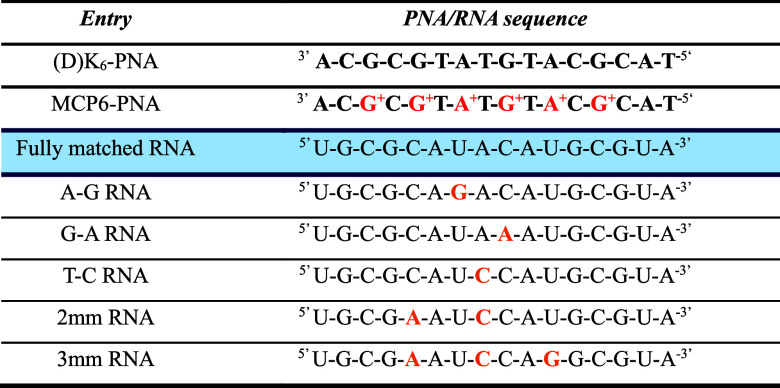
Synthetic RNA Sequences Used for Mismatch Discrimination Studies with MCP6 and (D)­K_6_ PNAs^a^

a Cationic purines are shown in red and mismatches in orange.

The fully matched (FM) RNA duplex exhibited the highest thermal stability, with *T*_m_ values of 71.0 °C for MCP6-PNA and 71.4 °C for the control (D)­K_6_–PNA sequence (Figure 5). Introducing a single mismatch opposite A^+^ or G^+^ reduced duplex stability, which was reflected by a decrease of approximately 6 °C in *T*_m_ values for both PNA sequences. A mismatch at the intervening position caused a slightly greater destabilization for MCP6-PNA (−7.9 °C) compared to the control (D)­K_6_–PNA (−6 °C).

**Figure 5:**
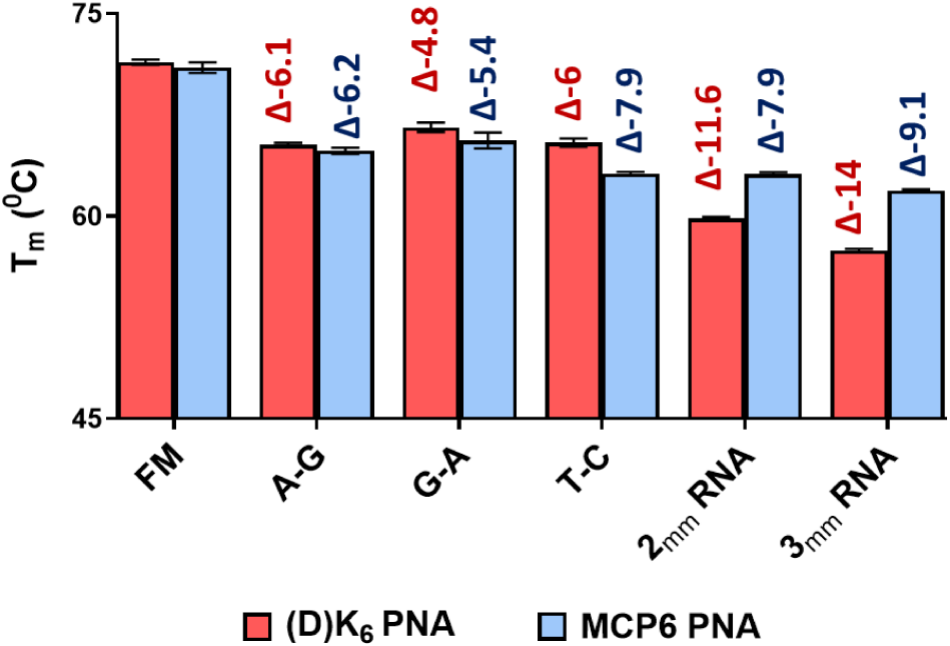
Thermal stability (*T*_m_) and mismatch discrimination of MCP6-PNA:RNA and control (D)­K_6_–PNA:RNA duplexes. Duplexes were hybridized with fully matched (FM) and mismatched RNA strands in PBS buffer. [PNA] = [RNA] = 2 μM.

Duplexes containing two or three mismatches showed a more pronounced loss in stability. The control (D)­K_6_–PNA displayed larger *T*_m_ reductions of 11.6 and 14.0 °C for the two- and three-mismatch duplexes, respectively, whereas MCP6-PNA exhibited only moderate decreases. This relative stabilization is likely due to electrostatic interactions between the positively charged purines in MCP6-PNA and the negatively charged RNA backbone, which partially offset the destabilizing effects of base mismatches. Importantly, MCP6-PNA maintained high sensitivity toward single mismatches, confirming that the introduction of positively charged purines preserves its sequence discrimination capability, particularly, for single nucleotide variants­(SNVs).

### Serum Stability and Cellular Evaluation in a Melanoma Cell Line (MeWo)

PNA is well-known for its exceptional stability in biological fluids, attributed to its resistance to enzymatic degradation.^64,65^ To assess whether the incorporation of multiple positive charges affects this property, MCP6-PNA and the control (D)­K_6_–PNA were incubated in fetal bovine serum (FBS) at 37 °C for 5 and 16 h (Figures S19–S20). Samples were collected at each time point and PNA integrity was analyzed by HPLC to follow degradation or potential adduct formation. A serum-only control was included to eliminate background interference from serum components. Both PNAs retained well-defined chromatographic peaks over 16 h, with no evidence of degradation or formation of secondary products. These findings indicate that the introduction of multiple positively charged purines in a PNA sequence does not compromise its stability under serum conditions.

To further examine whether MCP-PNAs, and particularly MCP6-PNA, demonstrate efficient uptake across different cell types, their internalization was evaluated in the melanoma cell line MeWo (Figure S21) under the same experimental conditions (2 μM, 37 °C, 5 h) used for OVCAR-8 cells. Flow cytometry analysis showed that MCP6-PNA displayed higher uptake than the other MCP-PNA variants, consistent with its behavior in OVCAR-8 cells. However, unlike in OVCAR-8, where MCP6-PNA showed the highest uptake among all PNAs, its internalization in MeWo cells was comparable to that of the CPP-conjugated controls (D)­K_4_–PNA and (D)­K_6_–PNA, while (D)­K_8_–PNA demonstrated the greatest overall uptake. Cell viability assays further confirmed that MCP-PNAs, including MCP6-PNA, did not cause detectable cytotoxicity in MeWo cells. Together, these results indicate that MCP6-PNA maintains efficient cellular entry across different cell types, though its relative performance is cell-line dependent, and importantly, it preserves high biocompatibility.

## Discussion

Since their initial description, PNAs have attracted considerable interest across diverse research fields due to their versatility in diagnostic and therapeutic applications. Their resistance to nuclease- and protease-mediated degradation in serum and cellular environments confers extended stability both in vitro and in vivo. However, limited cellular uptake has remained a major challenge for their use in intracellular sensing and antisense strategies, prompting extensive efforts to develop effective delivery approaches. These include nanocarrier-based systems such as liposomes and polymeric nanoparticles, chemical modifications, and formulation of PNAs as duplexes with sacrificial DNA to exploit established transfection technologies,^66^ as well as conjugation to functional moieties such as lipophilic groups, sugars, and cell-penetrating peptides.^67^

Given that unmodified PNAs exhibit poor cellular permeability, self-mediated cellular uptake generally requires the introduction of backbone or nucleobase modifications that promote membrane interaction or endocytosis.^67,68^

One such chemical approach relies on the introduction of a positive charge on the nucleobase without hampering DNA/RNA binding affinities. In this regard, to the best of our knowledge, there is only one company (OliPass PNA) that is advancing human clinical trials using multiple lipophilic and positively charged nucleobases (G, C, and A) on the PNA antisense sequence (https://www.olipass.com/front/eng/index.do).

Other synthetic approaches on PNA nucleobases, other than the *N*7-methylated guanine^58^ include a guanine-modified positively charged base (preQ1)^69^ and a double positively charged cytosine.^70^ In both reports, one to three modifications were introduced into a short PNA sequence (10-mer) and required multistep reactions.

In this study, we introduced *N*-methylated purines as nucleobase modifications within PNA sequences to confer cellular permeability without the need for CPP conjugation. MCP-PNAs were internalized by two distinct cancer cell lines—ovarian cancer cells (OVCAR-8; Figures 1 and 2) and melanoma cells (MeWo; Figure S21)—without detectable cytotoxicity (Figures S27–S28 and data not shown). Among the constructs examined, MCP6-PNA consistently exhibited the highest cellular uptake in both cell lines. Notably, while MCP6-PNA displayed uptake comparable to CPP-conjugated controls in MeWo cells, it outperformed all other PNA variants in OVCAR-8 cells. The differing uptake profiles observed for PNA-CPP conjugates as well as MCP4-PNA and MCP8-PNA across the two cell lines suggest that cellular internalization may be influenced by cell-line-dependent factors. This would imply that a screen of multiple charges on a given MCP-PNA sequence would be desirable to find the optimal number of positive charges for a given cell line or indication. The observation that MCP6-PNA enters cells better than other MCP-PNAs (including MCP8-PNA) is anticipated as such a behavior has been documented for positively charged CPP-PNAs.^71^ In a study by Abes and coworkers, the CPP with 6 Arg’s had better uptake into HeLa pLuc705 cells than a CPP-PNA with 3 or 9 arginines.

As MCP-PNAs contain multiple positive charges, it is not surprising that these PNAs are partially entrapped in lysosomes (Figure 3, MCP6-PNA). Nonetheless, not all MCP6-PNA is confined to the lysosome, suggesting that some may be bioavailable in the cytoplasm.

The enhanced cellular uptake observed for MCP-PNAs was not accompanied by appreciable changes in structural or thermal stability upon hybridization with complementary RNA (Figure S18 and Table 1). In contrast to reports by Aiba et al., where incorporation of one or three *N*-methylated guanine (G^+^) residues led to a measurable increase in PNA–DNA duplex melting temperature,^58^ MCP-PNAs displayed duplex stability toward complementary synthetic RNA similar to that of unmodified PNAs. Although MCP8-PNA, containing eight cationic purines, exhibited the highest melting temperature among the modified and unmodified constructs, this increase was modest.

Previously reported M^+^-modified PNAs have demonstrated enhanced cellular permeability together with increased binding affinity toward dsRNA targets.^56,57^ However, unlike M^+^ -modified PNAs, which showed improved cellular uptake relative to unmodified PNAs but did not reach the efficiency observed for PNA-CPP conjugates,^56^ MCP6-PNA exhibited uptake comparable to or exceeding that of unmodified CPP-PNAs (Figure 1 and Figure S21).

Whereas many backbone and alternative nucleobase modifications require synthetically demanding multistep procedures, *N*-methylation of purine nucleobases provides a one-step modification starting from commercially available PNA monomers. In particular, *N*-methylated guanine PNA monomers could be readily prepared in high yield, whereas the corresponding adenine modification was obtained in lower yield, indicating that further optimization may be required to improve its synthetic efficiency.

Despite significant advances in carrier-assisted PNA delivery, many existing platforms suffer from recurring challenges, including synthetic and formulation complexity, limited biodegradability, potential toxicity, constraints on PNA loading and release, as well as limited stability of peptide-based systems in biological environments and their potential immunogenicity.^66,72^ Moreover, delivery vectors such as cell-penetrating peptides, nanoparticles, or aptamers often rely on endocytic internalization pathways,^66,73^ resulting in endosomal entrapment that restricts access to intracellular targets and limits biological activity. This can be overcome by conjugation to membrane-active fusogenic peptides,^74^ attachment of reactive oxygen species (ROS) sensitizers that induce endosomal membrane disruption,^75^ or by the use of endosomolytic agents (e.g., chloroquine (CQ)^76^ or Ca2+^77^ ). These strategies frequently introduce additional complexity and potential toxicity.

Taken together, these findings demonstrate that incorporation of *N*-methylated purines provides a straightforward and accessible strategy for enhancing PNA cellular uptake while preserving duplex stability and avoiding the use of external delivery vectors. Although the mechanism of MCP-PNA internalization remains to be determined and will be addressed in future studies, the performance of MCP6-PNA across multiple cell lines highlights the potential of this approach and its relevance for advancing PNA-based applications.

## Conclusions

In this work, we show that incorporation of *N*-methylated purine nucleobases enables the generation of cell-permeable PNAs without the use of additional delivery components. Importantly, this nucleobase-level modification preserves the sequence-selective recognition properties of PNA, including sensitivity to single-nucleotide mismatches. Among the constructs examined, MCP6-PNA consistently exhibited the highest cellular uptake across multiple mammalian cell lines, underscoring the importance of charged nucleobase content in promoting cellular internalization.

Overall, these findings highlight nucleobase methylation as a simple and modular design element for improving PNA cellular uptake while maintaining target specificity and avoiding the complexity associated with carrier-assisted delivery approaches. This approach expands the scope of PNA chemical design and provides a useful platform for further development of PNA-based systems for intracellular nucleic acid recognition and antisense applications.

## Supplementary Material



## Notes

Characterization of PNAs; melting temperature (*T*_m_) measurements; assessment of mismatch sensitivity with RNA and DNA; serum stability of MCP6 and (D)­K_6_ PNAs; CD measurements of MCP6 and (D)­K_6_ PNAs; FACS analysis of MeWo cells; confocal microscopy of OVCAR-8 cells; characterization of A^+^ PNA monomer (PDF)

## Abbreviations

PNA: Peptide Nucleic Acid
MCP: Multiple Charged Purines
CPP: Cell-Penetrating Peptide
OC: Ovarian Cancer
