# Supplementary material for: Multiple Charged Purine (MCP) PNA as a Simple Mode for Cellular Uptake
Source: ACS Polym Au. 2026 Apr 21;6(3):881–90. doi: 10.1021/acspolymersau.6c00012 (PMC13261727; doi:10.1021/acspolymersau.6c00012)
Supplement: Supplementary file 1 [file lg6c00012_si_001.pdf]

Supporting Information

Multiple Charged Purine (MCP) PNA as a Simple Mode for Cellular Uptake

Salam Maree[1] and Eylon Yavin[1,*]

[1]The Institute for Drug Research, The School of Pharmacy, The Faculty of Medicine, The Hebrew University of Jerusalem, Hadassah Ein-Kerem, Jerusalem 9112102, Israel. E-mail: eylony@ekmd.huji.ac.il

Supporting Information

Tables of contents:

**General procedures and materials**	**3-4**
**HPLC and MS analysis of PNAs (Figure S1-S9)**	**5-9**
**Tm measurements (Figure S10-S17, Table 1)**	**9-14**
**Circular dichroism (CD) spectroscopy (Figure S18)**	**15**
**Serum Stability (Figure S19-S20)**	**16**
**Flow Cytometry Analysis (Figures S21-S25)**	**17-25**
**Confocal Microscopy (Figure S26)**	**26-27**
**Cell Viability (Figures S27-S28)**	**28-31**
**Synthesis and Characterization of A^+ PNA monomer (^1H, ^{13}C NMR spectra and HRMS) (Figures S29-S30, Scheme 1)**	**32-34**
**References**	**35**

General procedures and materials

Manual solid-phase synthesis was conducted using 5 mL polyethylene syringe reactors equipped with fritted disks (Phenomenex, Torrance, CA, USA). HPLC purifications and analysis were carried out on a Dionex UltiMate 3000 HPLC system (ThermoFisher Scientific, Waltham, MA, USA), employing a semi-preparative C18 reversed-phase column (Jupiter C18, 10 µm, 300 Å, 250 × 10 mm, Phenomenex) with automated fraction collection. Eluents: A (0.1% TFA in water) and B (MeCN) were used in a linear gradient with a flow rate of 4mL/min. Mass spectrometric analysis of PNAs was performed using MALDI-TOF MS (Microflex LRF, Bruker Daltonics) with 2,5-dihydroxybenzoic acid (DHB) as the matrix.
RNA oligo was purchased from IDT, USA. Fmoc-protected PNA monomers were purchased from PolyOrg, Inc. (USA) and used without further purification. Additional reagents, including Fmoc-D-(tBOC)-Lys(OH), Rhodamine B, and Fmoc-PEG$_2$-CH$_2$COOH, as well as other materials required for solid-phase synthesis, were procured from Merck (Germany) and Biolab (Israel). Positively charged purines (G^+ and A^+) were synthesized as previously reported.[1]

Solid phase synthesis of PNAs

Coupling of Fmoc-D-(tBOC)-Lys(OH) onto Novasyn TGA Resin. The resin (100 mg, 0.25 mmol/g) was was swelled in 2 mL of DMF for 2 hours. For pre-activation, a solution containing 10 equivalents of Fmoc-D-(tBOC)-Lys(OH) (0.25 mmol, 117 mg) in DCM (2.5 mL) was cooled in an ice bath, followed by the addition of diisopropylcarbodiimide (DIC, 5 equiv., 0.125 mmol, 15.8 mg, 19.5 µL) and 4-dimethylaminopyrimidine (DMAP, 0.1 equiv., 0.0025 mmol, 0.3 mg). After 20 min, the mixture was evaporated, re-dissolved in dry DMF and added to the pre-swelled resin. After 5h, the resin was washed with dichloromethane (5x2 mL), DMF (5x2 mL). The coupling procedure was then repeated to ensure complete loading.

Fmoc Deprotection. The Fmoc group was removed by treating the resin twice with 20% piperidine in DMF for 10 minutes (×2), followed by washing with DCM (5x2 mL) and DMF (5x2 mL).

Coupling of Fmoc-Bhoc-PNA-Monomers, Fmoc-Bhoc-G$^+$-monomer, Fmoc-Bhoc-A$^+$-monomer and Fmoc-PEG$_2$-CH$_2$COOH. For a 10 µmols scale synthesis on TGA-NovaSyn resin (loading−0.25 mmol/g), Fmoc-protected amino acids, Fmoc-Bhoc-PNA monomers or Fmoc-PEG$_2$-CH$_2$COOH (40 µmol) were activated in dry DMF (0.4 mL) with 2-(1H-7-

azabenzotriazol-1-yl)-1,1,3,3-tetramethyluronium hexafluorophosphate methanaminium (HATU, 40 µmol, 15.2 mg), hydroxybenzotriazole (HOBt, 40 µmol, 5.4 mg), and N,N-diisopropylethylamine (DIPEA, 80 µmol, 14 µL). After 5 min of pre-activation, the mixture was added to the resin and allowed to react for 60 minutes. The resin was then washed sequentially with DCM (5 × 2 mL) and DMF (5 × 2 mL) before proceeding to the next coupling step.

Coupling of Rhodamine B. For synthesis on a 10 µmol scale using TGA-NovaSyn resin (loading: 0.25 mmol/g), a mixture containing 4 equivalents of Rhodamine B (40 µmol), 4 eq. of HATU (40 µmols, 15.2 mg), 4 eq. of HOBT (40 µmols, 5.4 mg) and 8 eq. of dry DIPEA (80 µmols, 14µL), in DMF (to 0.1 M PNA) were mixed in a glass vial equipped with a screw cap. After 5 min of pre-activation, the solution was transferred to the resin and allowed to react for overnight. Following the coupling, the reaction mixture was removed, and the resin was washed sequentially with DCM (5 × 2 mL) and DMF (5 × 2 mL).

Cleavage of PNA from resin. Deprotection and cleavage of the PNA–peptide conjugates from the resin were performed using a cleavage cocktail consisting of trifluoroacetic acid (TFA) and m-cresol (90:10, v/v). The resin was treated twice with 1 mL of the cleavage mixture for 2 hours each. The PNAs were precipitated by trituration with cold diethyl ether, followed by centrifugation and decantation of the supernatant. The residues were dissolved in water and purified by semi preparative HPLC. The purified PNAs were characterized by MALDI-TOF mass spectrometry.

HPLC and MS of MCPs and control PNAs

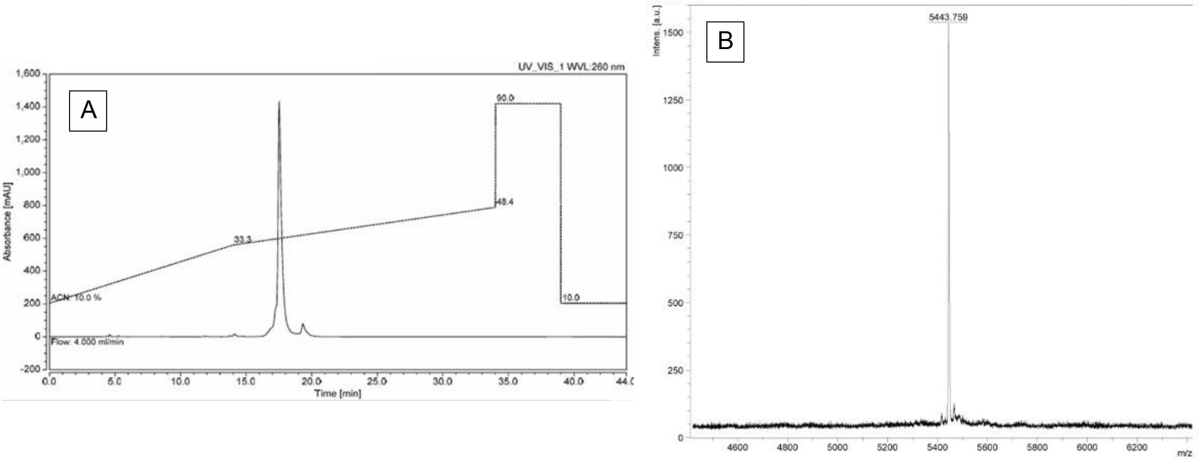

Figure S1: Characterization of (D)K_4 PNA (control PNA). (A) HPLC chromatogram. Eluents: A (0.1% TFA in water) and B (MeCN) were used in a linear gradient (10-33.3 % B over 14 min, 33.3-48.4% over 20 min) with a flow rate of 4 mL/min and elution at 17.5 min. **(B)** Maldi-TOF MS. M_{calc} = 5437.6, M_{obs} =5443.7.

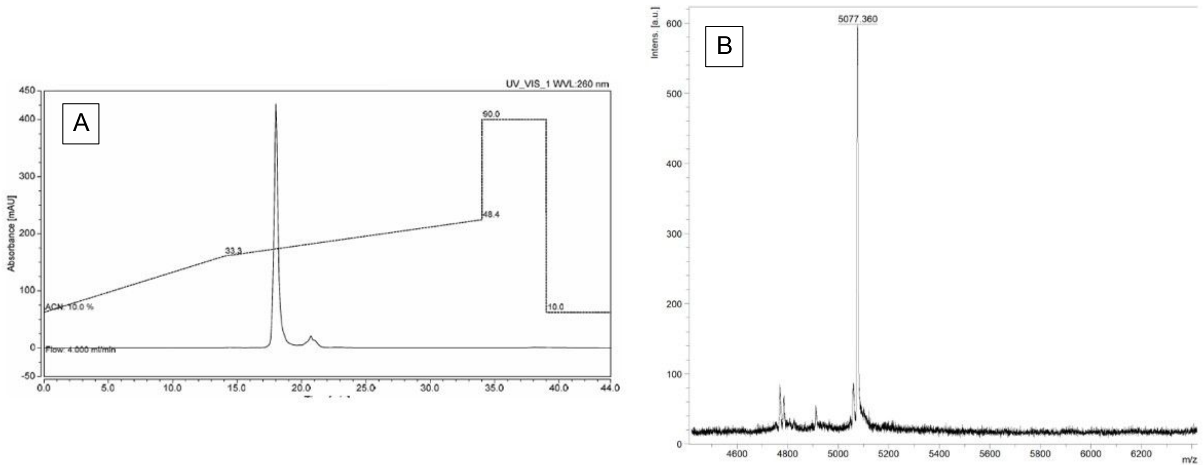

Figure S2: Characterization of MCP4 PNA. (A) HPLC chromatogram. Eluents: A (0.1% TFA in water) and B (MeCN) were used in a linear gradient (10-33.3 % B over 14 min, 33.3-48.4% over 20 min) with a flow rate of 4 mL/min and elution at 18 min. **(B)** Maldi-TOF MS. M_{calc} 5079.1 $[M+Na]^+$, M_{obs} =5077.3.

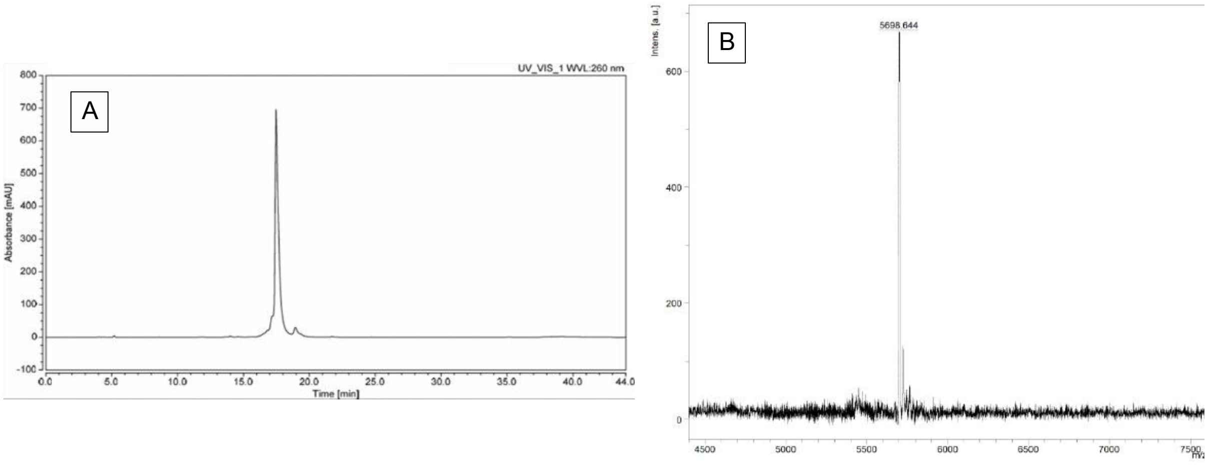

Figure S3: Characterization of (D)K_6 PNA (control PNA). (A) HPLC chromatogram. Eluents: A (0.1% TFA in water) and B (MeCN) were used in a linear gradient (10-33.3 % B over 14 min, 33.3-48.4% over 20 min) with a flow rate of 4 mL/min and elution at 17.5 min. **(B)** Maldi-TOF MS. M_{calc} = 5693.9, M_{obs} =5698.6.

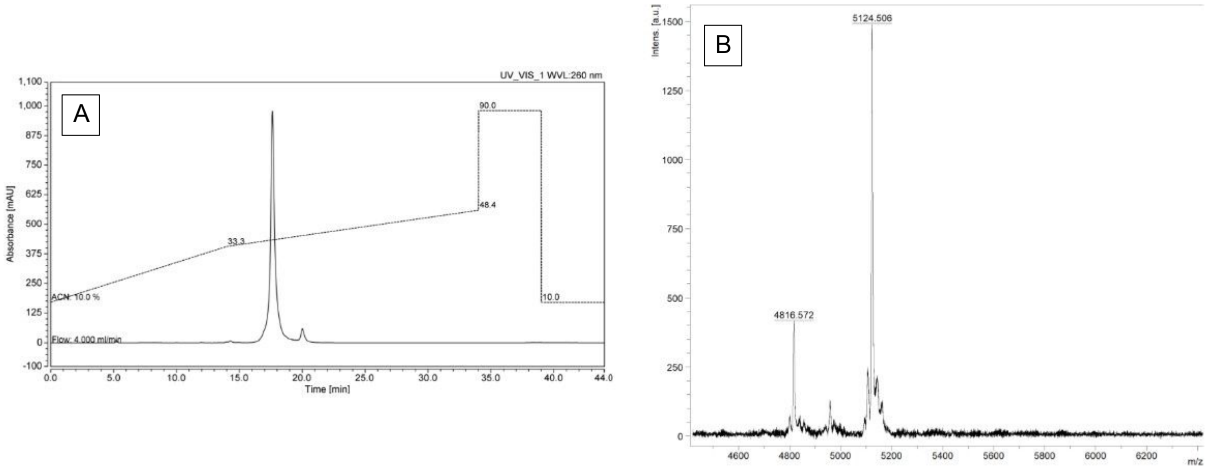

Figure S4: Characterization of MCP6 PNA. (A) HPLC chromatogram. Eluents: A (0.1% TFA in water) and B (MeCN) were used in a linear gradient (10-33.3 % B over 14 min, 33.3-48.4% over 20 min) with a flow rate of 4 mL/min and elution at 17.5 min. **(B)** Maldi-TOF MS. M_{calc} =5124.3$[M+k]^+$, M_{obs} =5124.5.

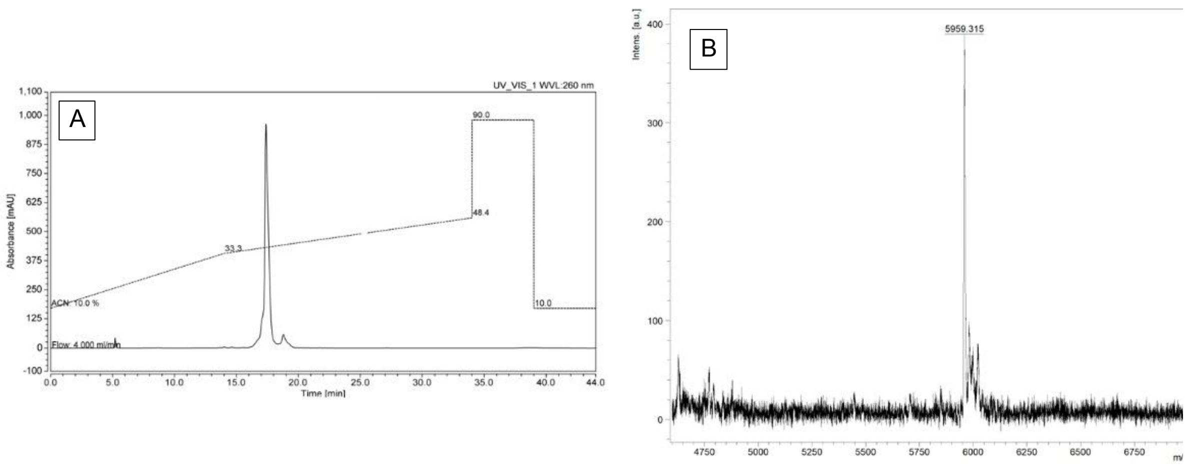

Figure S5: Characterization of (D)K$_8$ PNA (control PNA). (A) HPLC chromatogram. Eluents: A (0.1% TFA in water) and B (MeCN) were used in a linear gradient (10-33.3 % B over 14 min, 33.3-48.4% over 20 min) with a flow rate of 4 mL/min and elution at 17.5 min. **(B)** Maldi-TOF MS. M_{calc} = 5950.3, M_{obs} =5959.3.

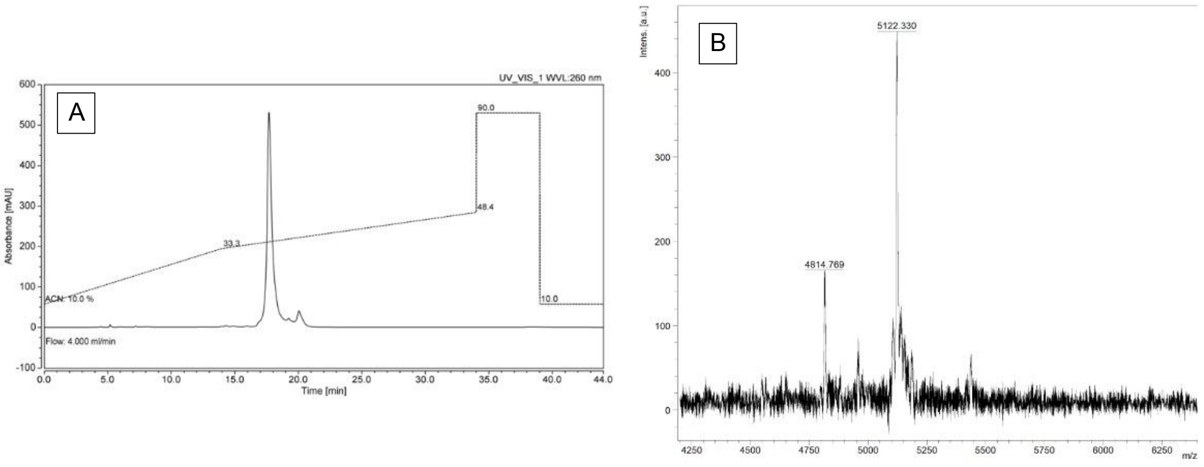

Figure S6: Characterization of MCP8 PNA. (A) HPLC chromatogram. Eluents: A (0.1% TFA in water) and B (MeCN) were used in a linear gradient (10-33.3 % B over 14 min, 33.3-48.4% over 20 min) with a flow rate of 4 mL/min and elution at 17.5 min. **(B)** Maldi-TOF MS. M_{calc}= 5116.2, M_{obs} =5122.3.

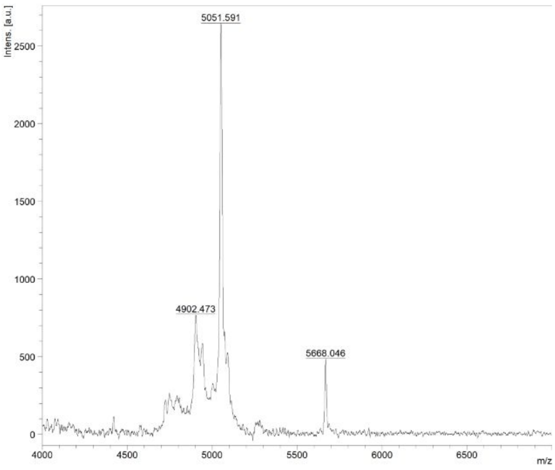

Figure S7: Maldi-TOF MS of the fully matched RNA. M_{calc}= 5081.1, M_{obs} =5051.59.

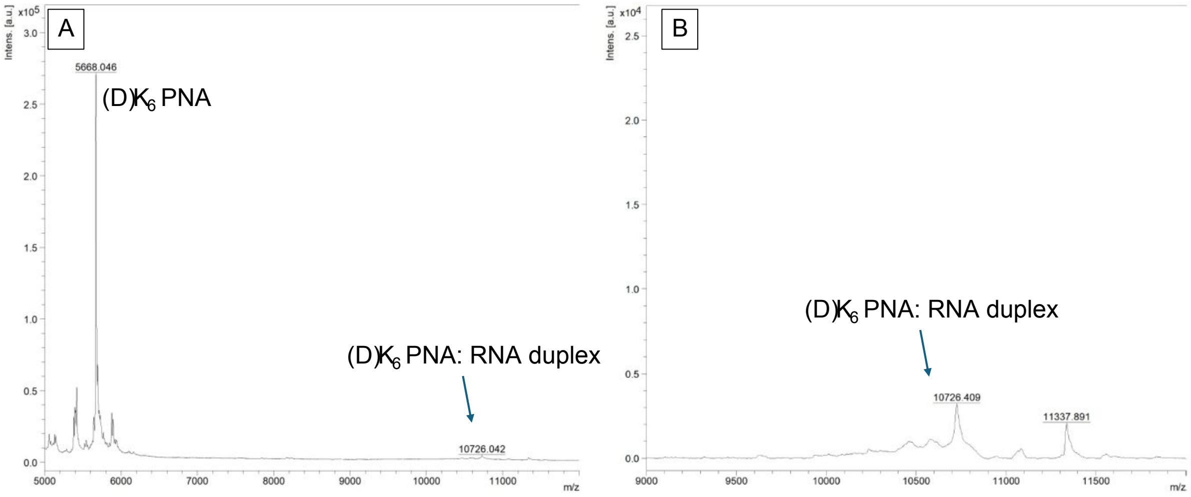

Figure S8: Maldi-TOF MS of (D)K_6 PNA: RNA duplex. (D)K_6 PNA M_{calc}= 5693.9, M_{obs} =5668.046. RNA M_{calc}= 5081.1, M_{obs} =5051.591. (D)K_6 PNA: RNA duplex M_{obs} =10726.409 (the sum of PNA and RNA values in Maldi-TOF MS=10719.6).

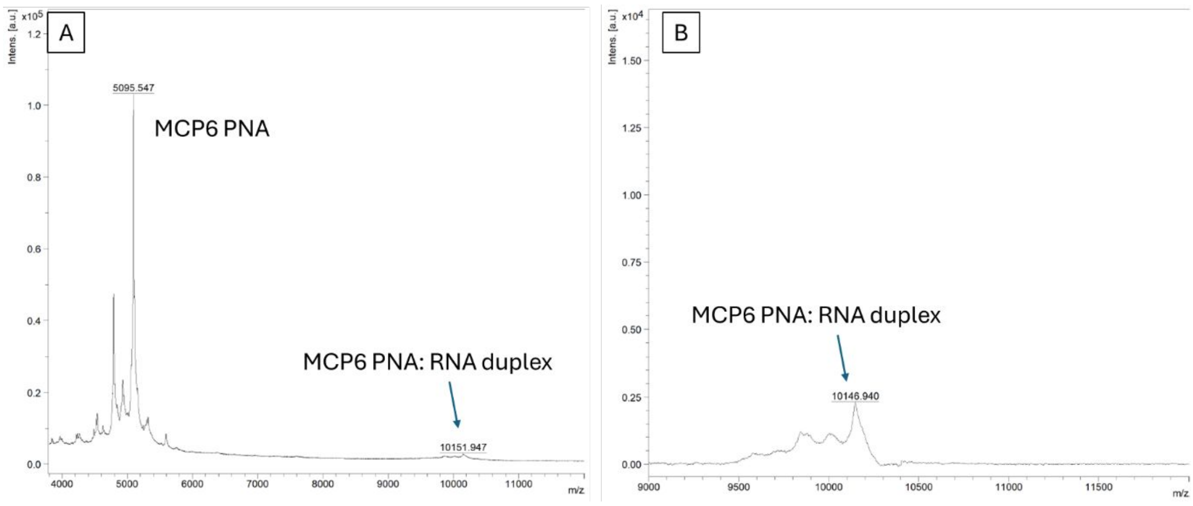

Figure S9: Maldi-TOF MS of MCP6 PNA: RNA duplex. MCP6 PNA M_{calc}= 5085.187, M_{obs} =5095.547. RNA M_{calc}= 5081.1, M_{obs} =5051.591. MCP6 PNA: RNA duplex M_{obs} =10146.94 ((the sum of PNA and RNA values in Maldi-TOF MS=10147.1).

Melting temperatures (Tm) measurement

Table S1. Tm measurements of control and MCP PNAs with complementary synthetic RNA.

Entry	(D)K_4 PNA	MCP4 PNA	(D)K_6 PNA	MCP6 PNA	(D)K_8 PNA	MCP8 PNA
T_m (^{0}C) of PNA: RNA	70 (±0.6)	69.3 (±0.2)	71.4 (±0.2)	71 (±0.4)	69.5 (±0.2)	73 (±0.3)

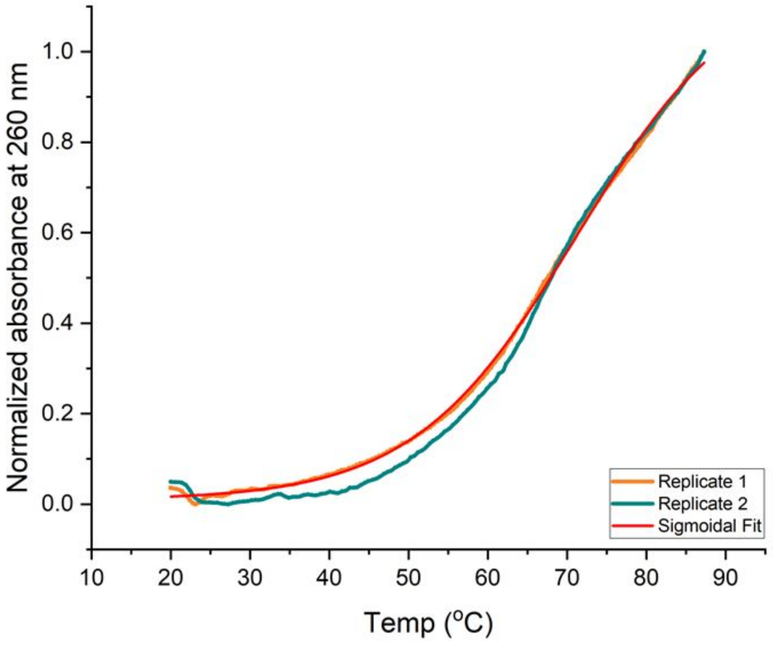

Figure S10: Representative melting curve profile for (D)K_4 PNA annealed to complementary synthetic RNA. [PNA] = [RNA] = 2µM.

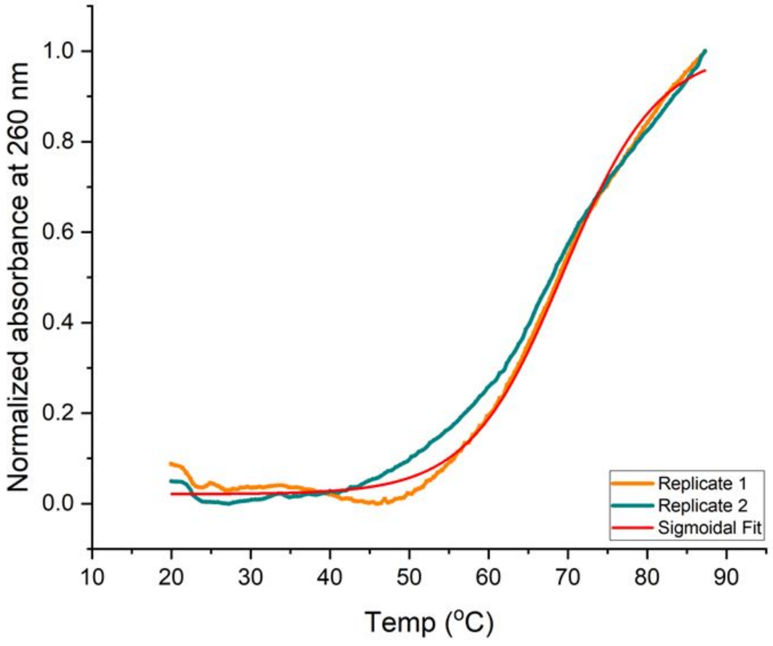

Figure S11: Representative melting curve profile for MCP4 PNA annealed to complementary synthetic RNA. [PNA] = [RNA] = 2µM.

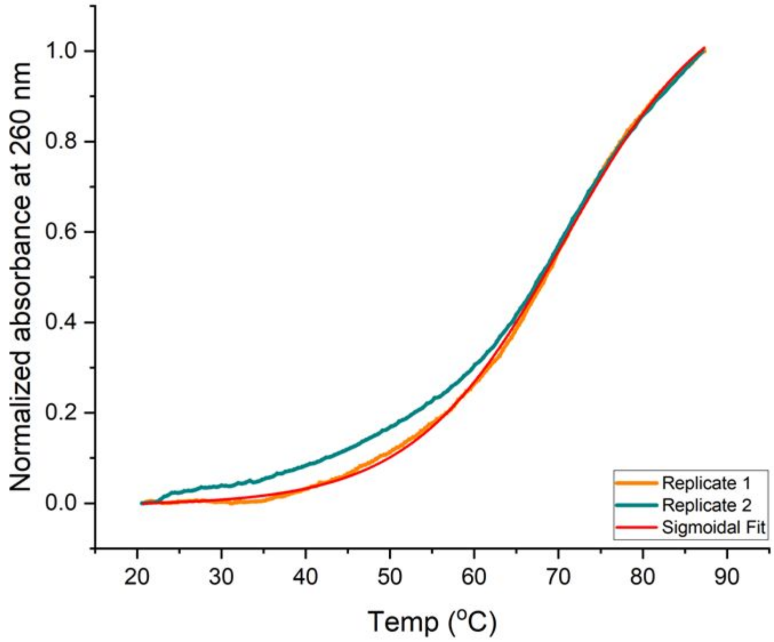

Figure S12: Representative melting curve profile for (D)K_6 PNA annealed to complementary synthetic RNA. [PNA] = [RNA] = 2μM.

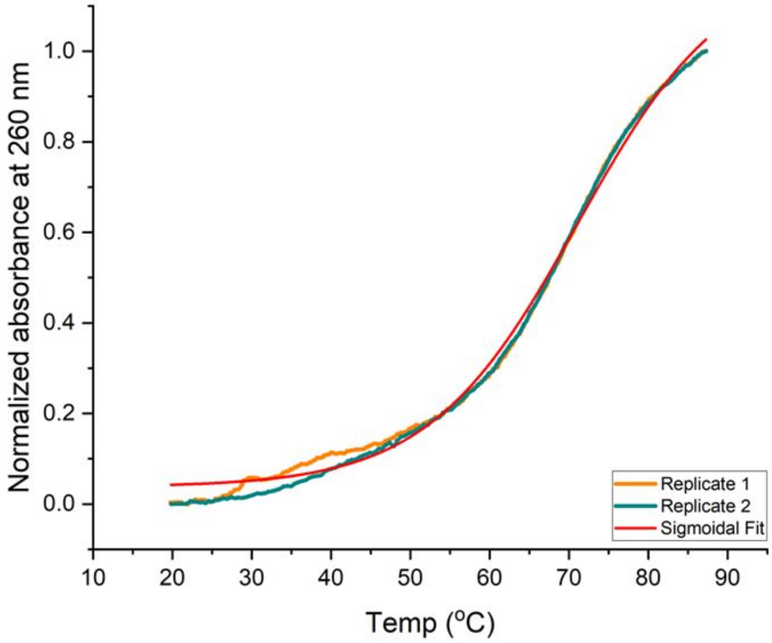

Figure S13: Representative melting curve profile for MCP6 PNA annealed to complementary synthetic RNA. [PNA] = [RNA] = 2μM.

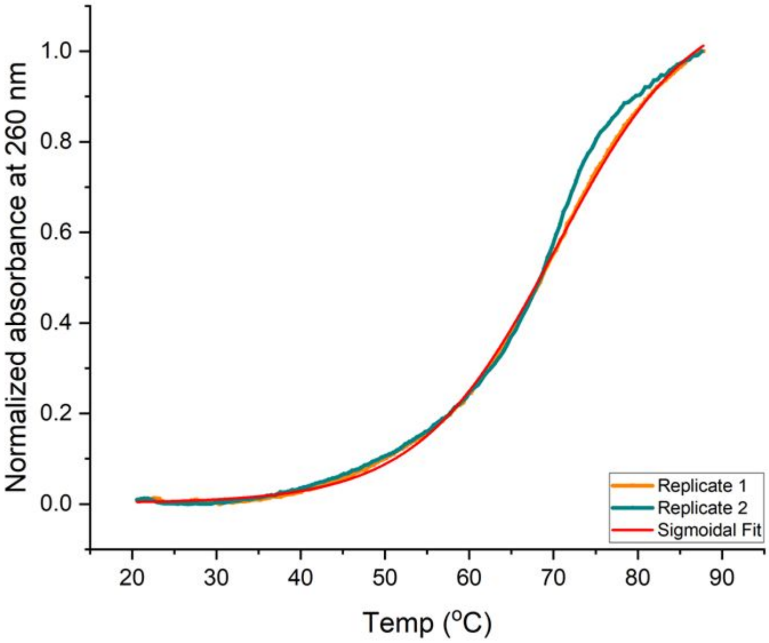

Figure S14: Representative melting curve profile for (D)K_8 PNA annealed to complementary synthetic RNA. [PNA] = [RNA] = 2μM.

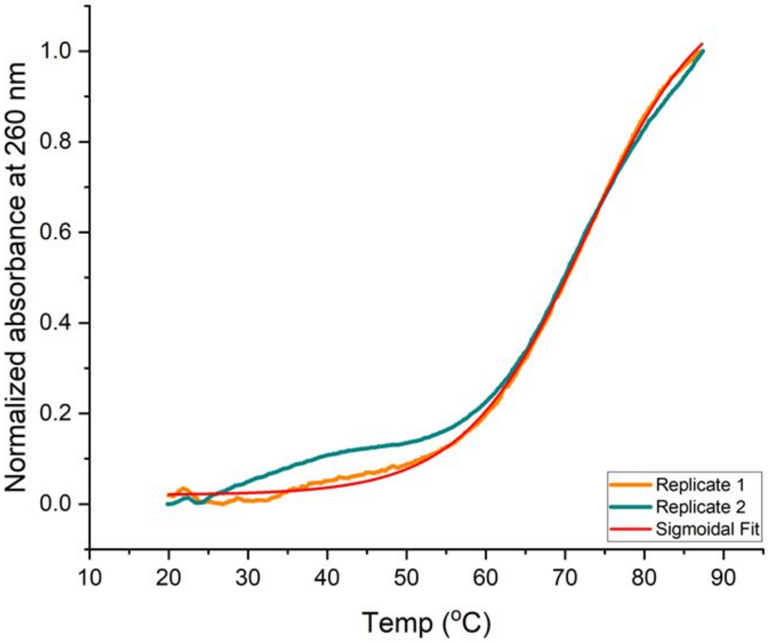

Figure S15: Representative melting curve profile for MCP8 PNA annealed to complementary synthetic RNA. [PNA] = [RNA] = 2μM.

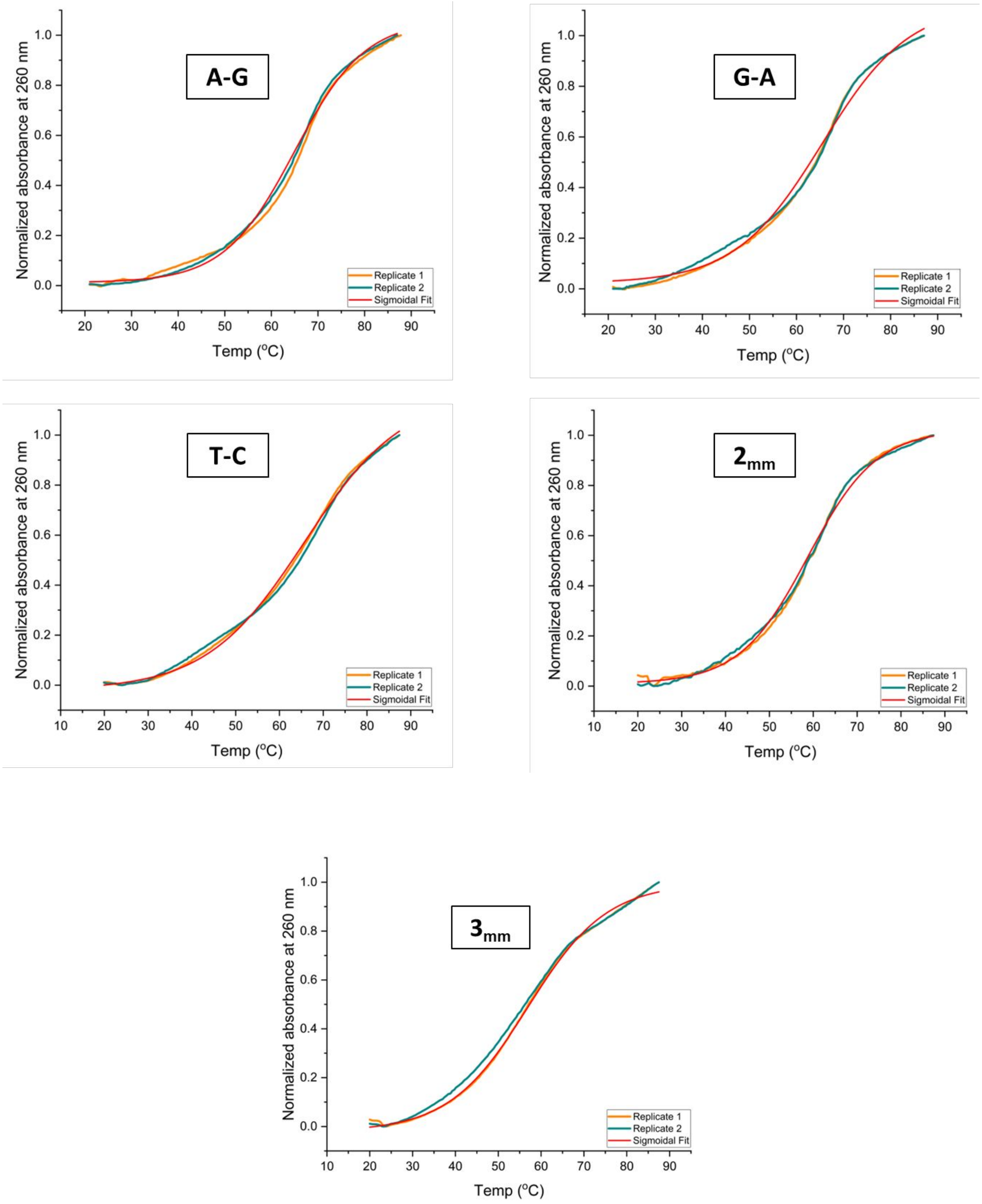

Figure S16: Melting curve profiles for (D)K_6 PNA annealed to mismatched synthetic RNA. [PNA] = [RNA] = 2μM.

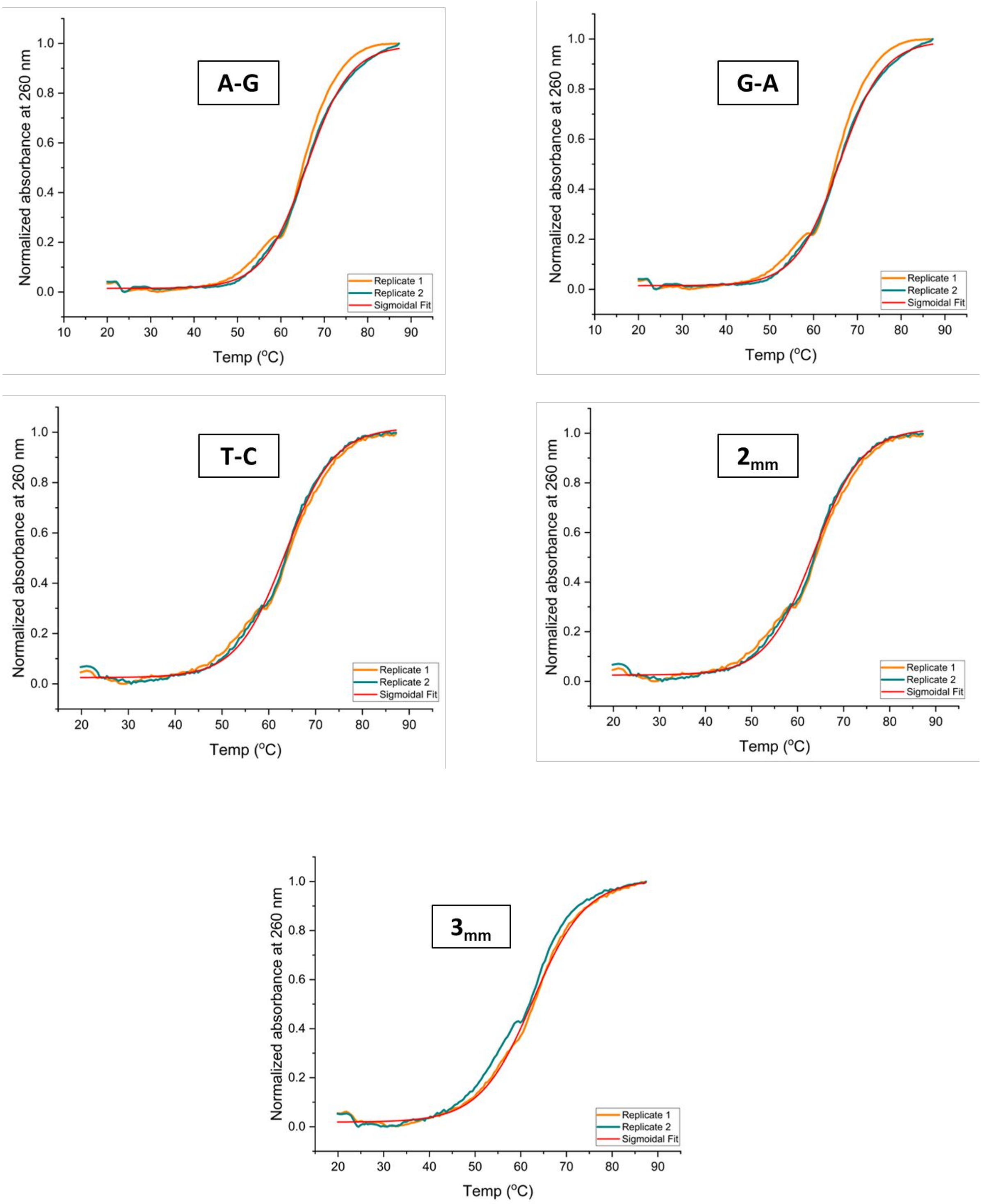

Figure S17: Melting curve profiles for MCP6 PNA annealed to mismatched synthetic RNA. [PNA] = [RNA] = 2μM.

Circular dichroism (CD) spectroscopy

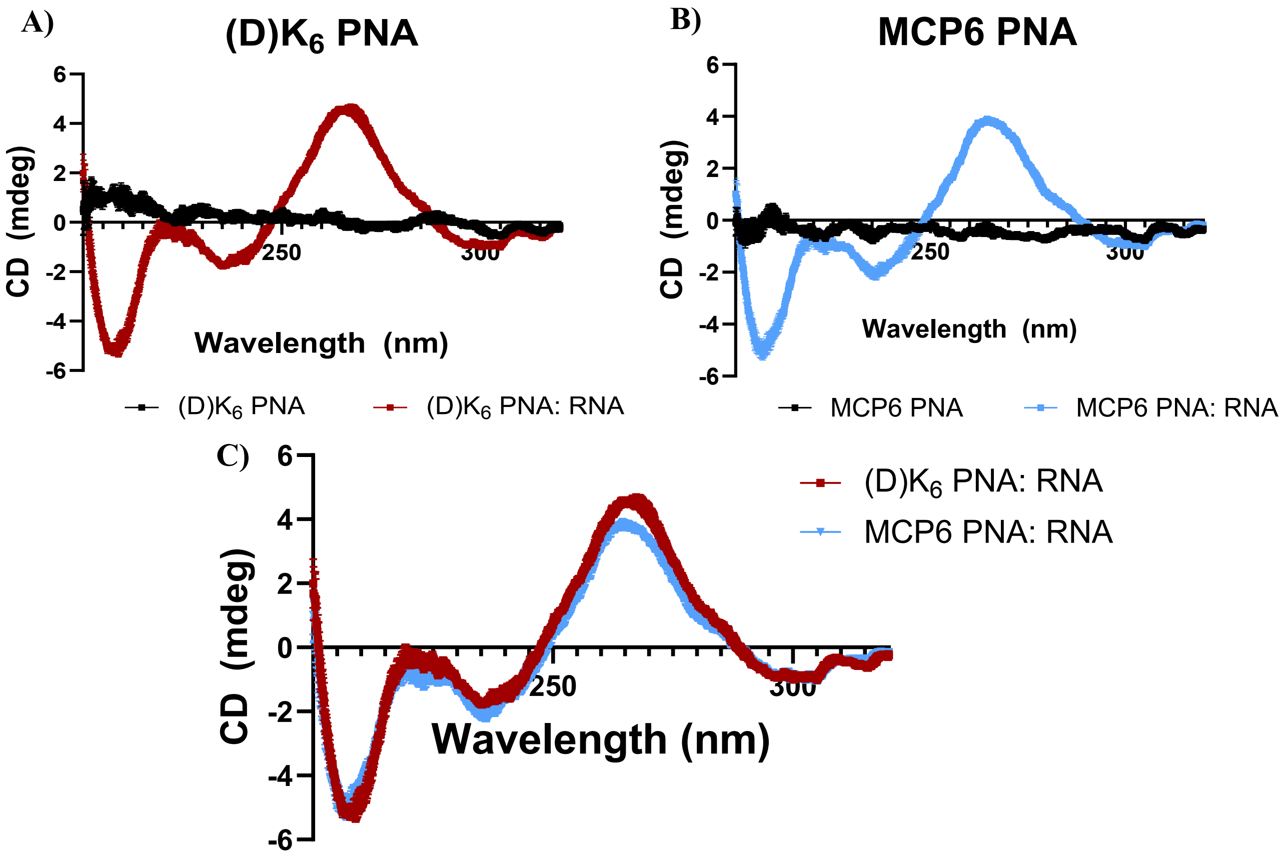

Fig. S18: CD spectra of (D)K6 and MCP6 PNA. **(A)** CD spectra of (D)K6 with and without the presence of complementary RNA in PBS buffer. **(B)** CD spectra of MCP6 with and without the presence of complementary RNA in PBS buffer. **(C)** Overlaid CD spectra of (D)K6 and MCP6 with complementary RNA in PBS buffer. [PNA] =[RNA]= 15μM.

Serum Stability

(D)K_6 and MCP6 PNAs were prepared at a final concentration of 20 μM in Fetal Bovine Serum (FBS) and incubated at 37 °C for 5, and 16 hours. The stability of the PNA-serum solutions was monitored at each time point in HPLC. A serum-only solution was used as a control.

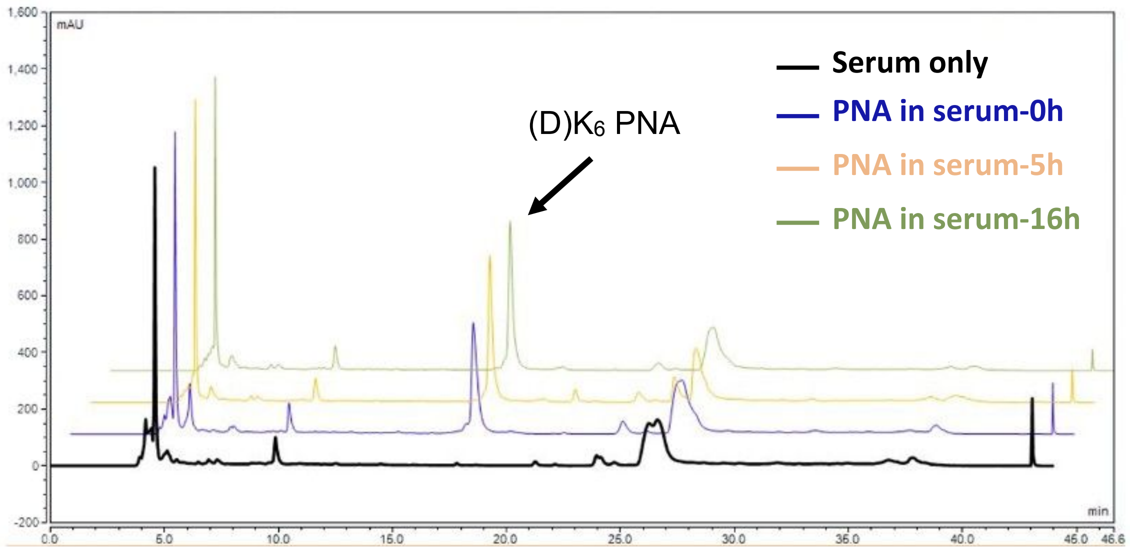

Figure S19: Serum stability of (D)K_6 PNA in FBS. PNAs were incubated with serum at 37 °C for 5 and 16 hours. [PNA]= 20 μM.

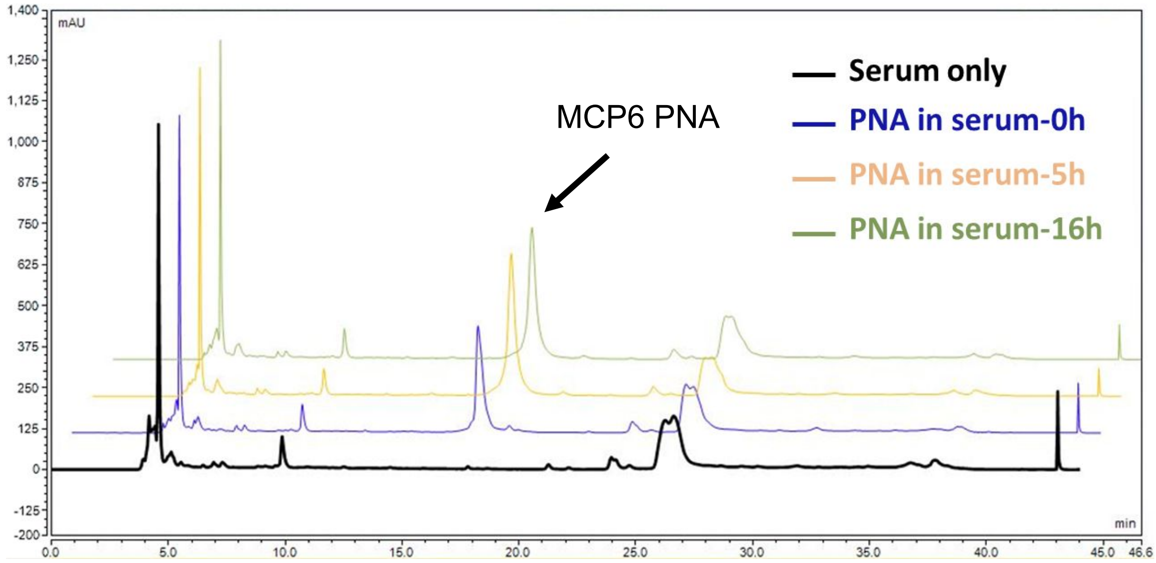

Figure S20: Serum stability of MCP6 PNA in FBS. PNAs were incubated with serum at 37 °C for 5 and 16 hours. [PNA]= 20 μM.

Flow Cytometry Analysis

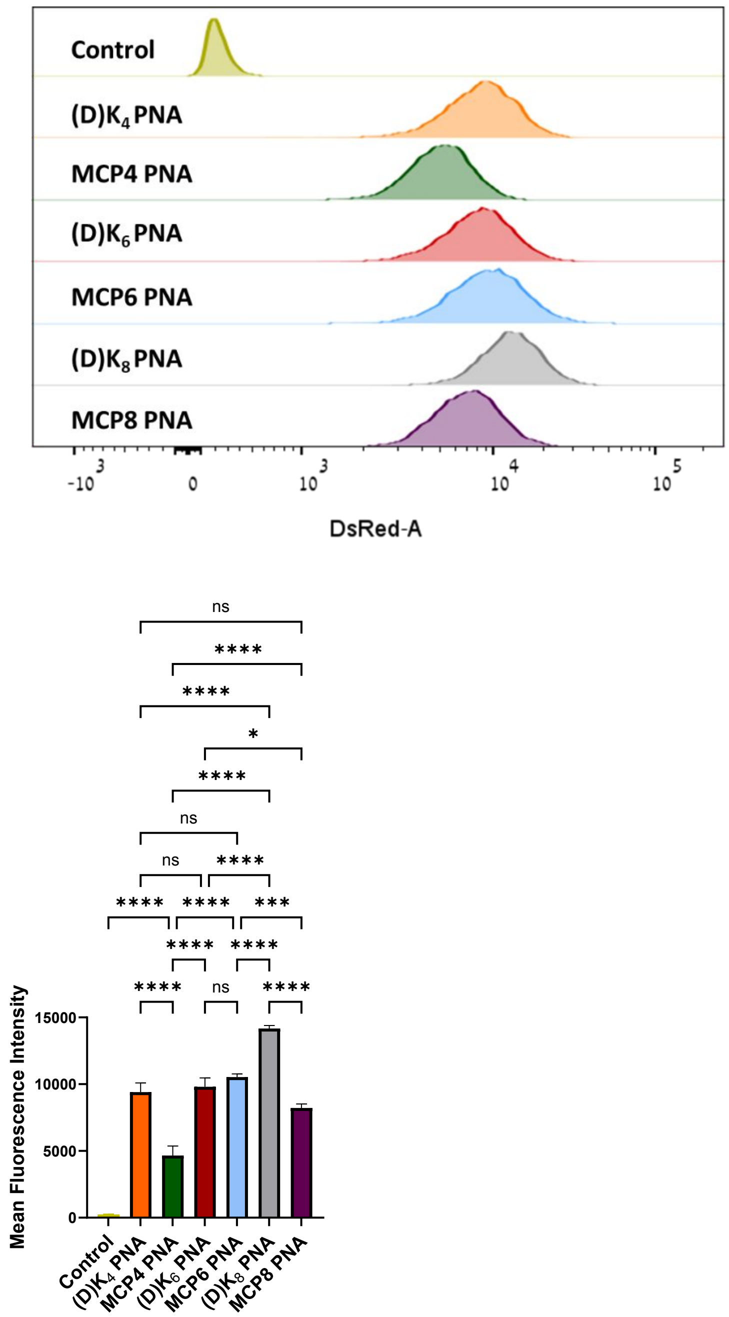

Figure S21: Flow cytometry analysis in MeWo cells after incubation with 2 μm of PNAs for 5h at 37 ^{0}C. Untreated cells served as control. (A) Histogram of FACS analysis in MeWo cells treated with PNAs. Histogram illustrates the mean fluorescence intensity plotted in horizontal axis against the number of cell events detected in the vertical axis. (B) Mean fluorescence intensity of PNAs in MeWo cells. The Data is presented as the mean ± SD (n = 3). *** represents $p \leq 0.001$, ** represents $p \leq 0.01$ and * represents $p \leq 0.05$ as determined by a One-way ANOVA test.

Supporting Information

A.

OVCAR-8
Control

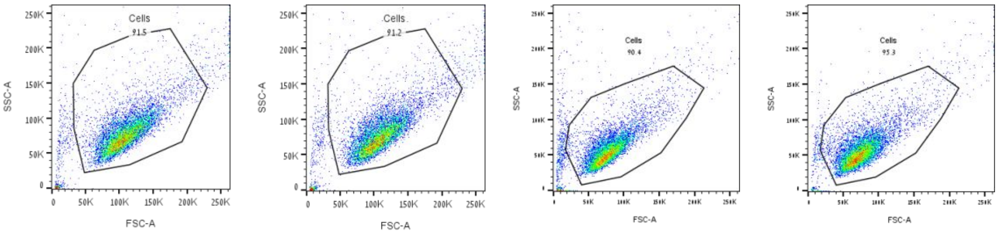

B.

OVCAR-8
(D)K_4 PNA

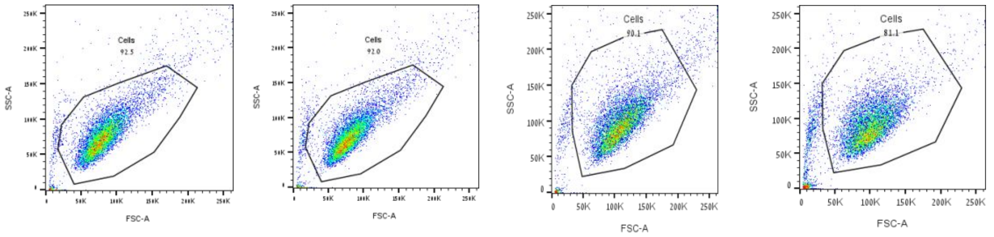

C.

OVCAR-8
MCP4 PNA

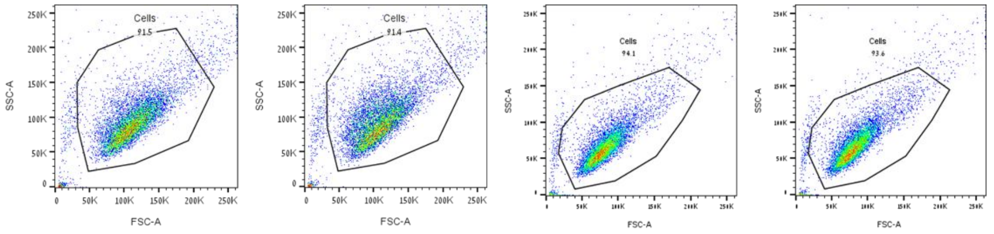

D.

OVCAR-8
(D)K_6 PNA

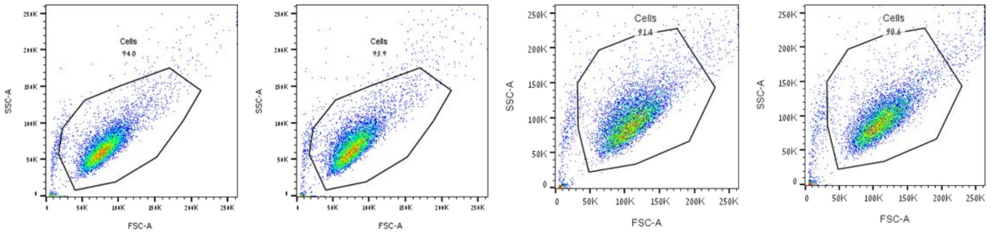

E.

OVCAR-8
MCP6 PNA

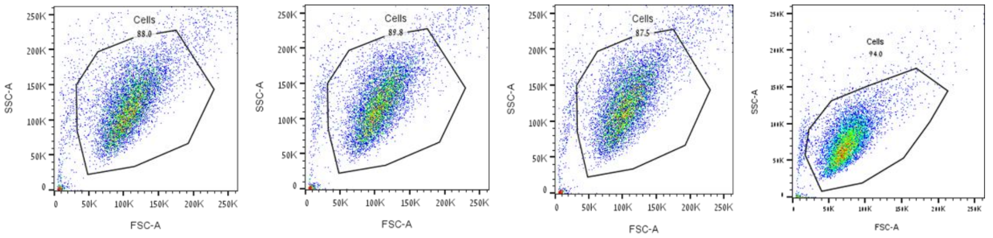

F.

OVCAR-8
(D)K_8 PNA

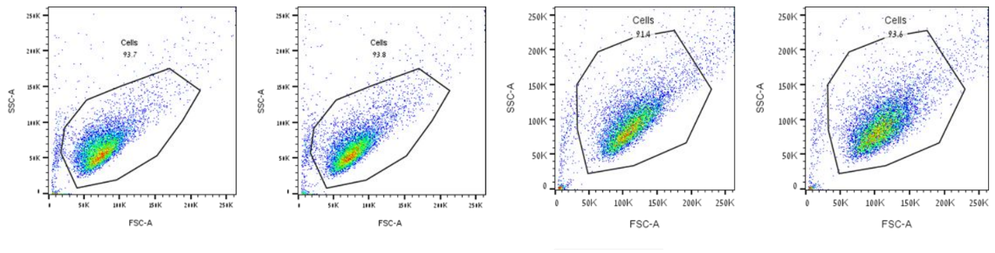

G.

OVCAR-8
MCP8 PNA

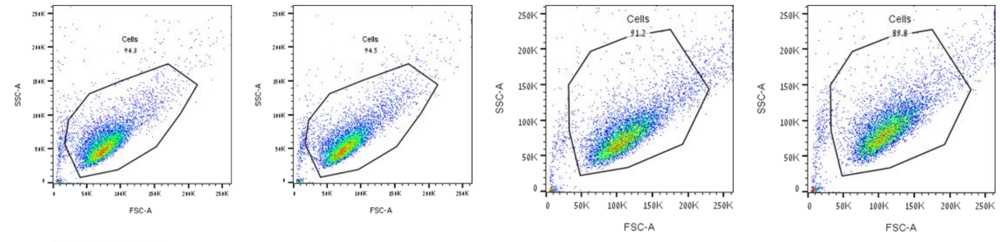

Figure S22: Forward and sideward scatter plots of OVCAR-8 cells incubated with 2 µM of PNAs for 5 h at 37°C in media. **(A)** Untreated OVCAR-8 cells (served as control), **(B)** (D)K_4 PNA, **(C)** MCP4 PNA, **(D)** (D)K_6 PNA, **(E)** MCP6 PNA, **(F)** (D)K_8 PNA and **(G)** MCP8 PNA.

Supporting Information

A.

OVCAR-8 - Control

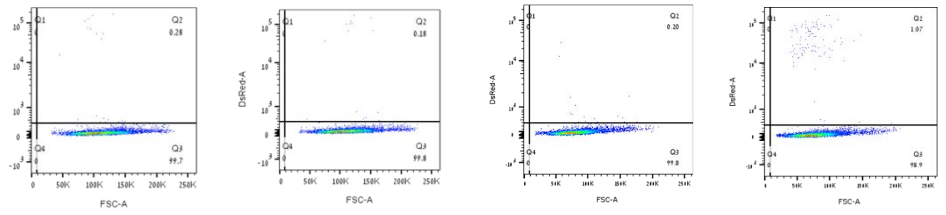

B.

OVCAR-8 - (D)K_4 PNA

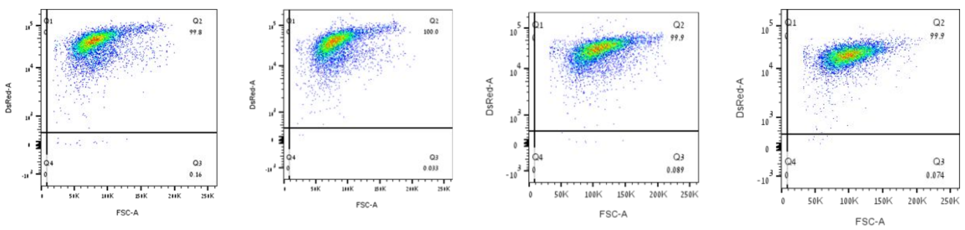

C.

OVCAR-8 - MCP4 PNA

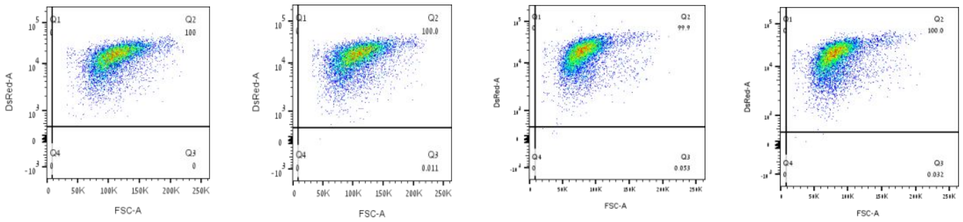

D.

OVCAR-8 - (D)K_6 PNA

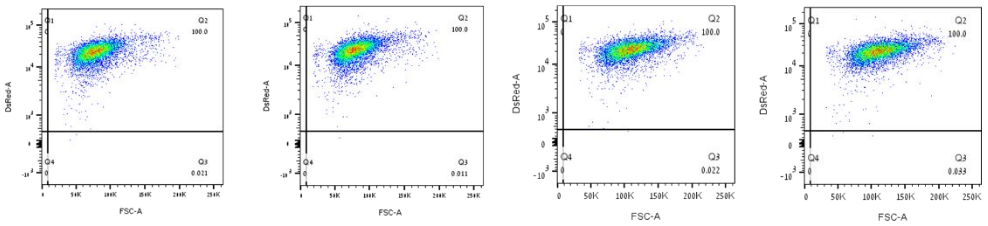

E.

OVCAR-8 - MCP6 PNA

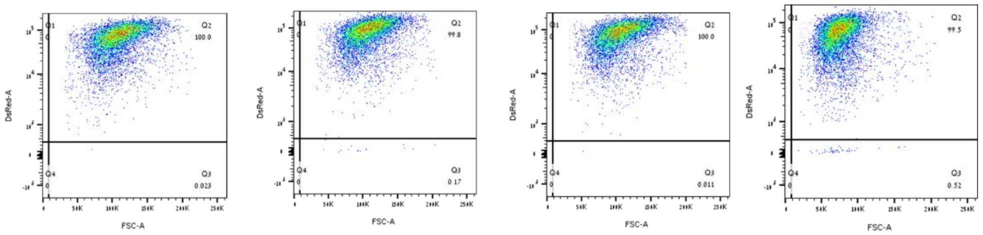

F.

OVCAR-8 -
(D)K_8 PNA

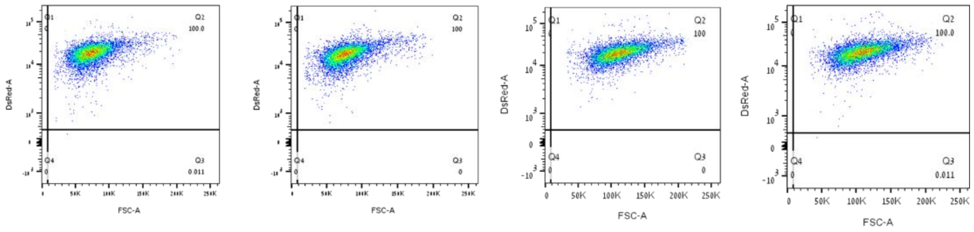

G.

OVCAR-8 -
MCP8 PNA

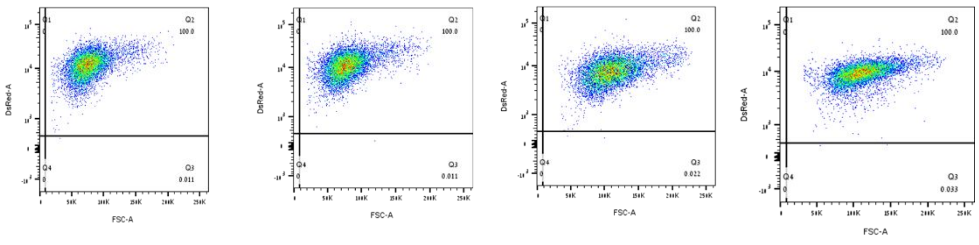

Figure S23: FACS results of replicates for PNAs in OVCAR-8 cells. The cells were incubated with 2 μM of PNA for 5 h at 37°C in media. The data is gated to the DsRed-A-positive populations. **(A)** Untreated OVCAR-8 cells (served as control), **(B)** (D)K_4 PNA, **(C)** MCP4 PNA, **(D)** (D)K_6 PNA, **(E)** MCP6 PNA, **(F)** (D)K_8 PNA and **(G)** MCP8 PNA.

Supporting Information

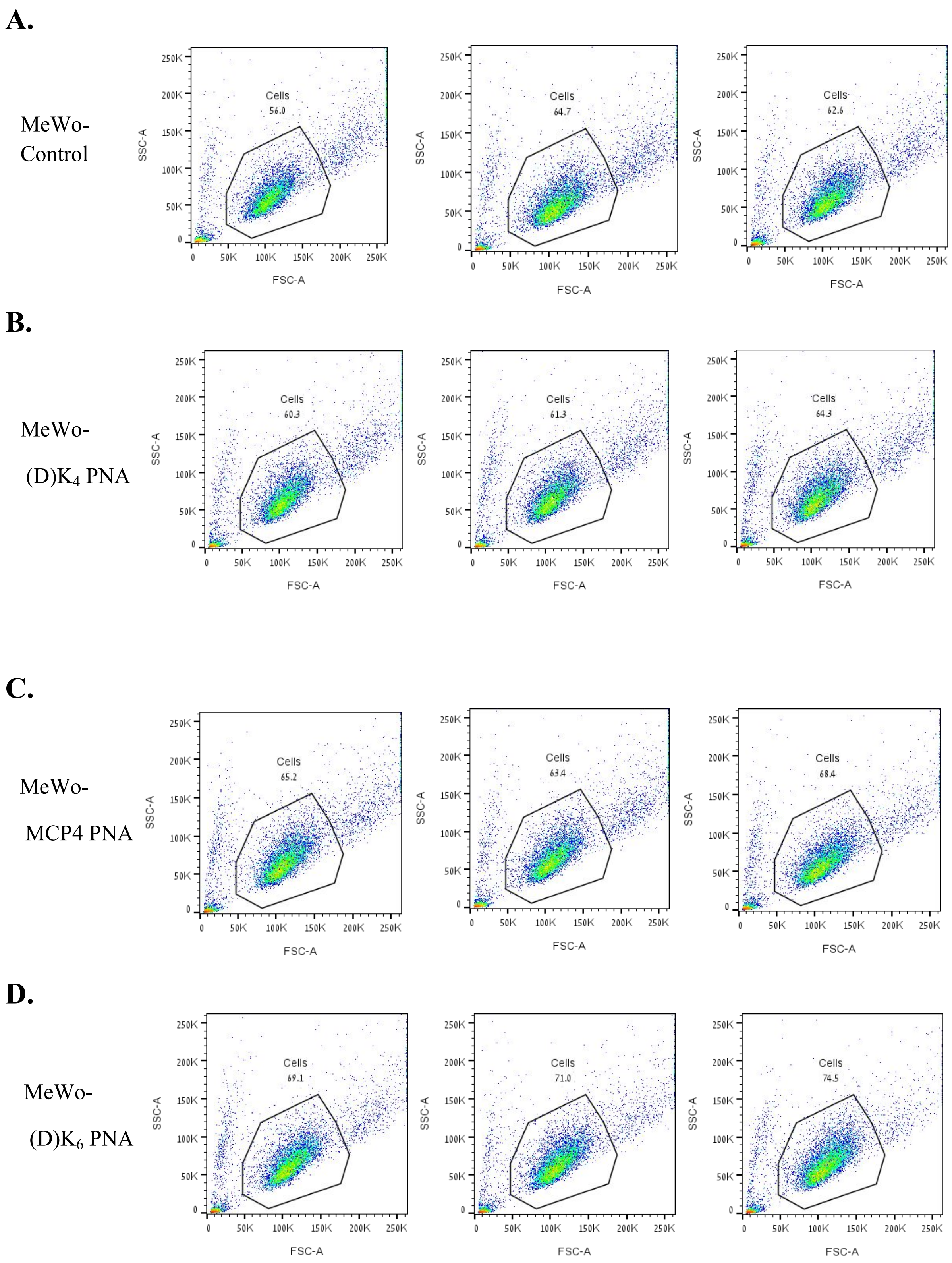

E.

MeWo-
MCP6 PNA

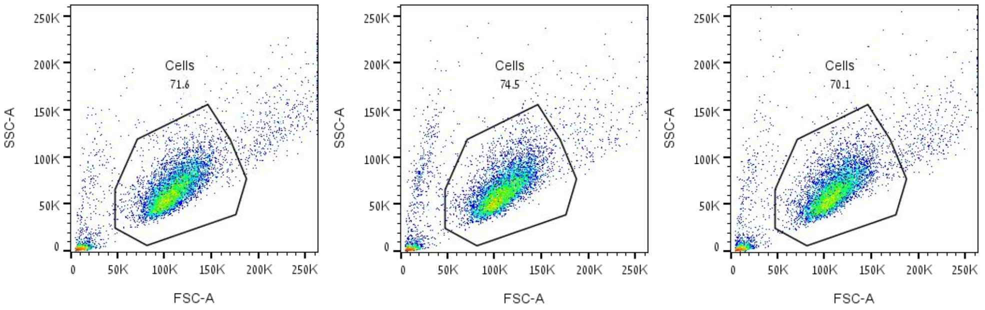

F.

MeWo-
(D)K_8 PNA

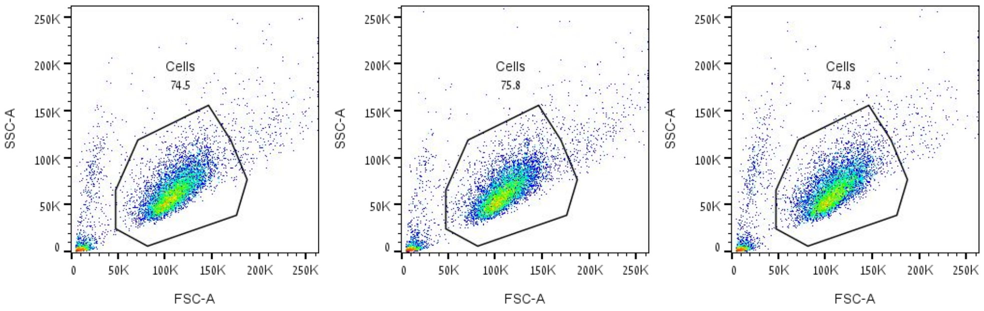

G.

MeWo-
MCP8 PNA

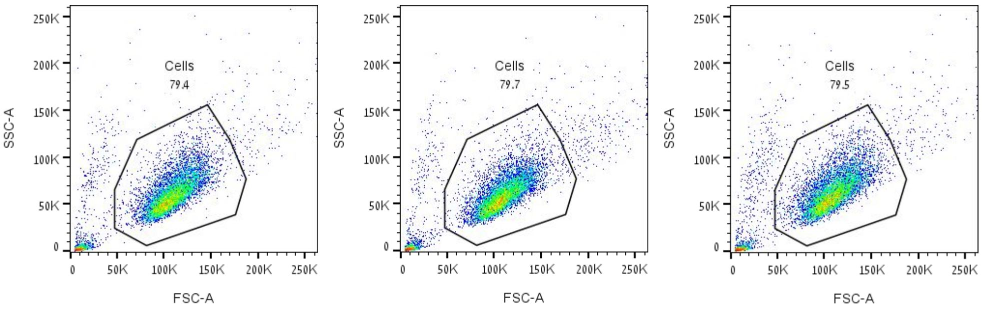

Figure S24: FACS results of replicates for PNAs in MeWo cells. The cells were incubated with 2 μM of PNA for 5 h at 37°C in media. **(A)** Untreated MeWo cells (served as control), **(B)** (D)K_4 PNA, **(C)** MCP4 PNA, **(D)** (D)K_6 PNA, **(E)** MCP6 PNA, **(F)** (D)K_8 PNA and **(G)** MCP8 PNA.

Supporting Information

A.

MeWo-
Control

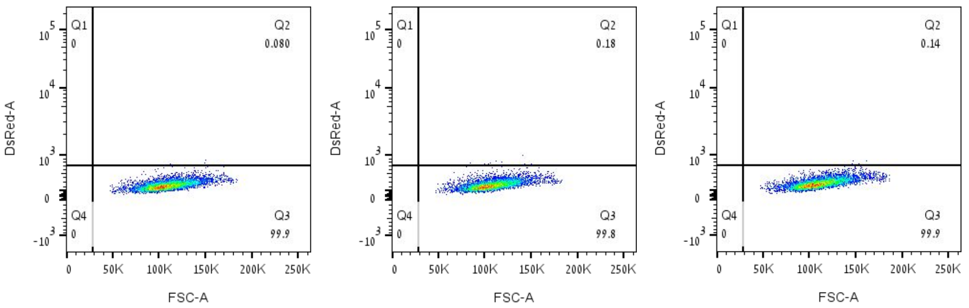

B.

MeWo-
(D)K_4 PNA

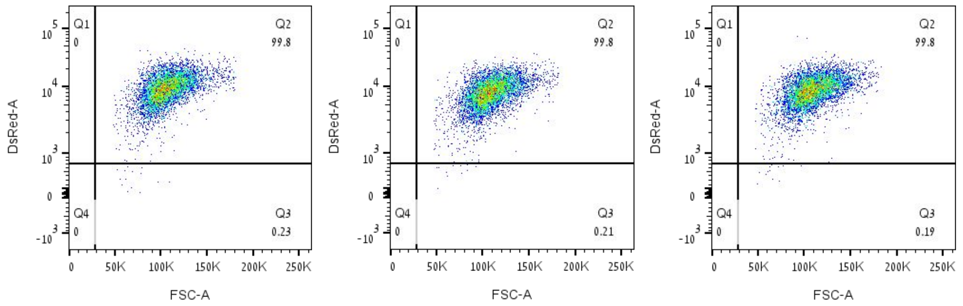

C.

MeWo-
MCP4 PNA

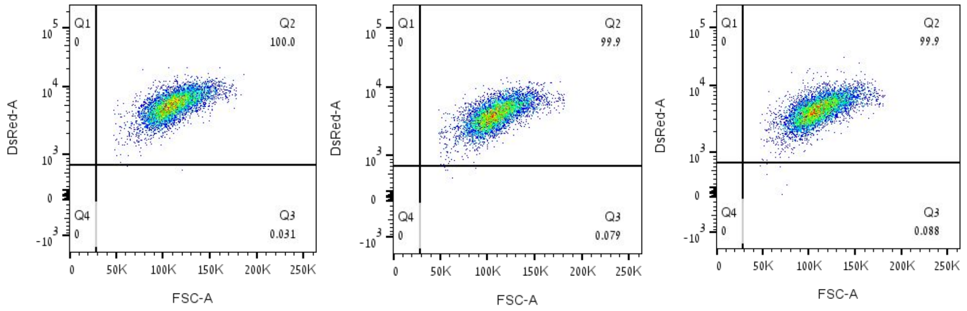

D.

MeWo-
(D)K_6 PNA

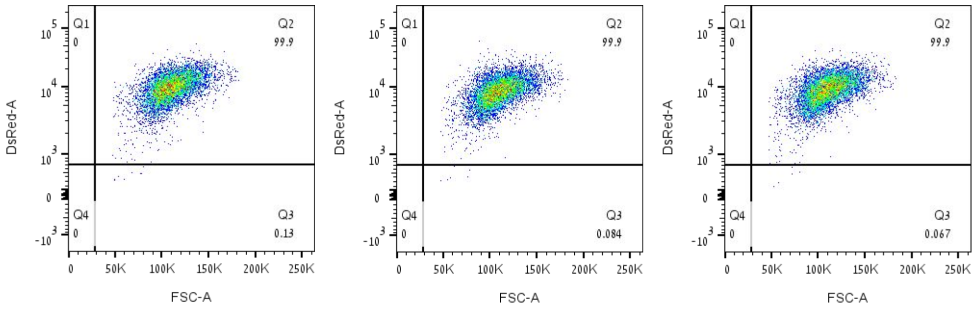

E.

MeWo- MCP8 PNA

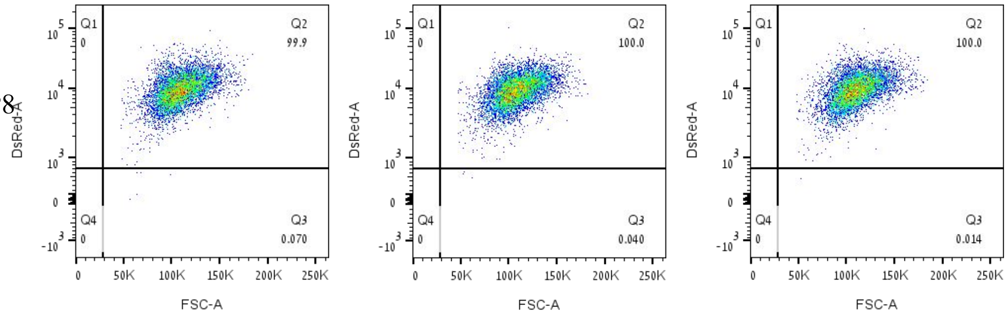

F.

MeWo-

(D)K_8 PNA

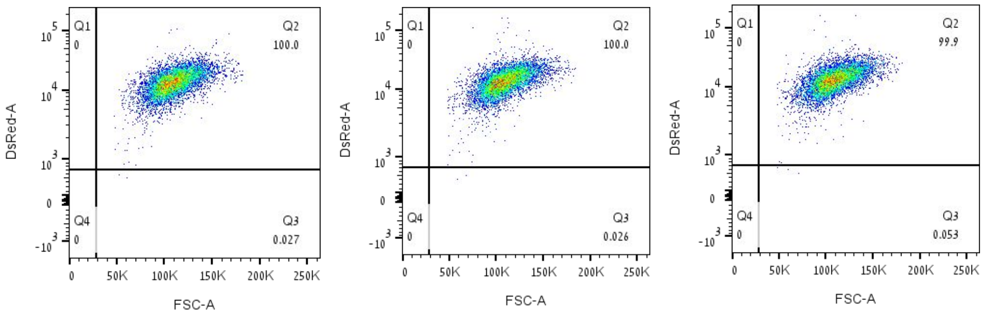

G.

MeWo-

MCP8 PNA

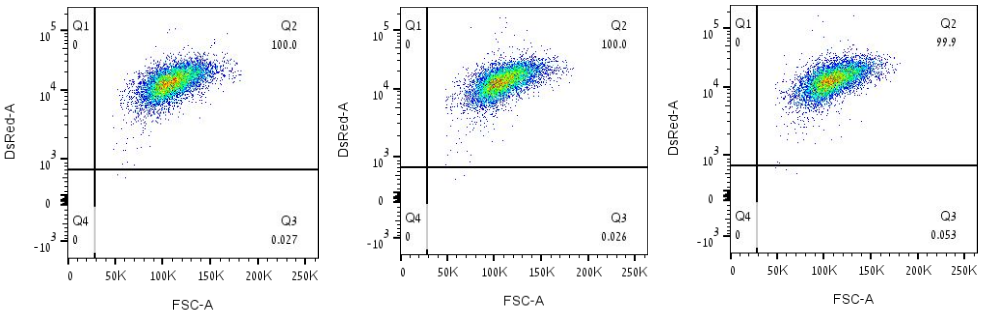

Figure S25: FACS results of replicates for PNAs in Mewo cells. The cells were incubated with 2 μM of PNA for 5 h at 37°C in media. The data is gated to the DsRed-A-positive populations. **(A)** Untreated MeWo cells (served as control), **(B)** (D)K_4 PNA, **(C)** MCP4 PNA, **(D)** (D)K_6 PNA, **(E)** MCP6 PNA, **(F)** (D)K_8 PNA and **(G)** MCP8 PNA.

Confocal Microscopy Images

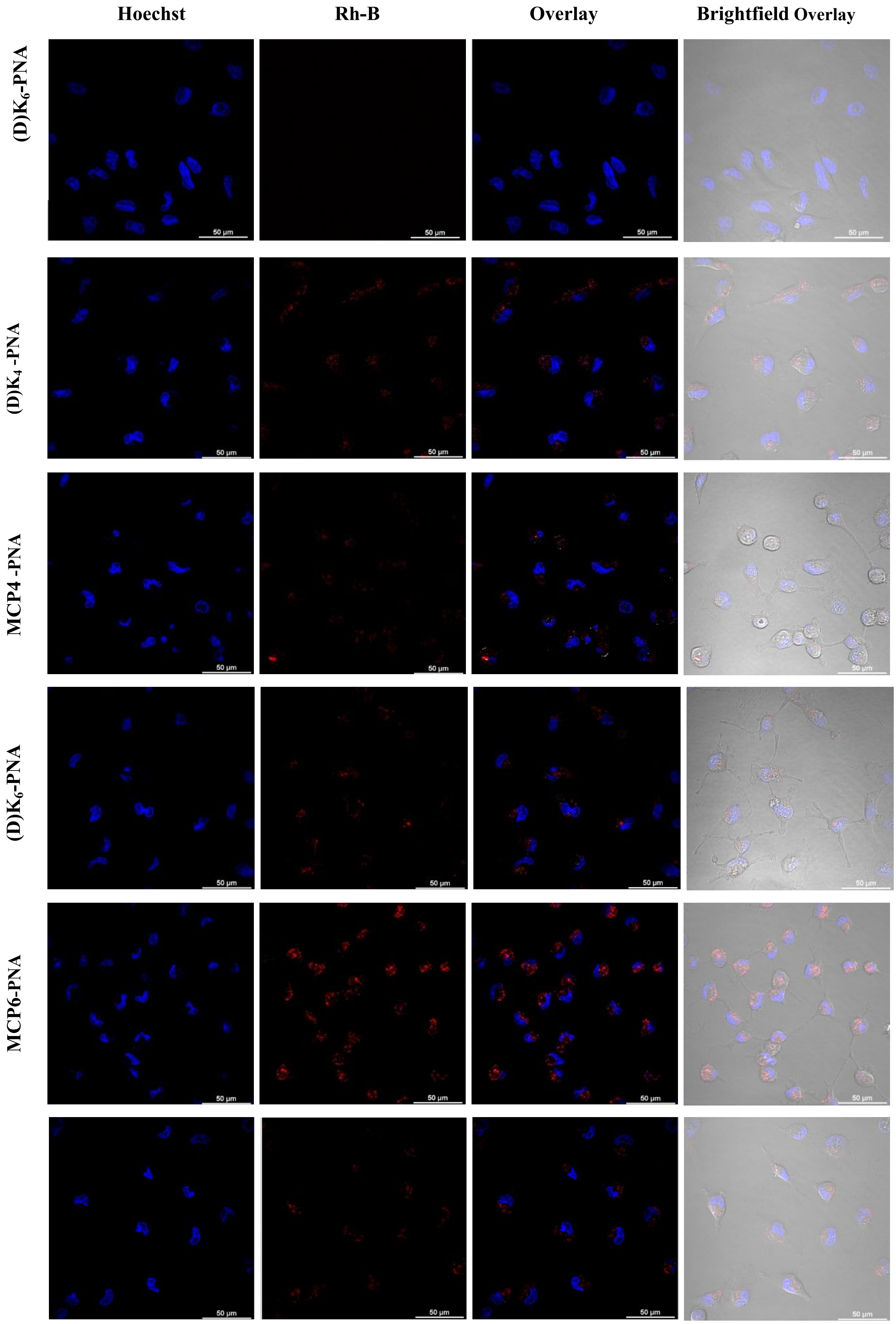

MCP8-PNA

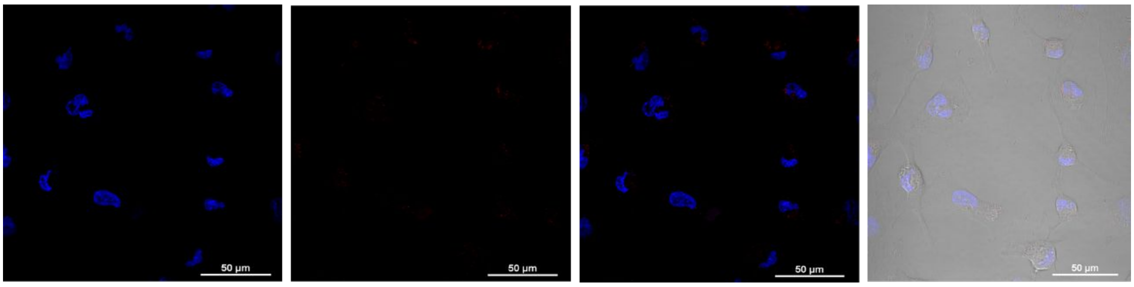

Figure S26: Confocal imaging of control and MCP PNAs (in red) in OVCAR-8 cells with the nucleus labelled by Hoechst marker (blue)**.** Scale bar = 50 µm. Cells were treated with 2 µM of PNAs for 5 h at 37°C. Untreated cells served as control.

Cell Viability Assay

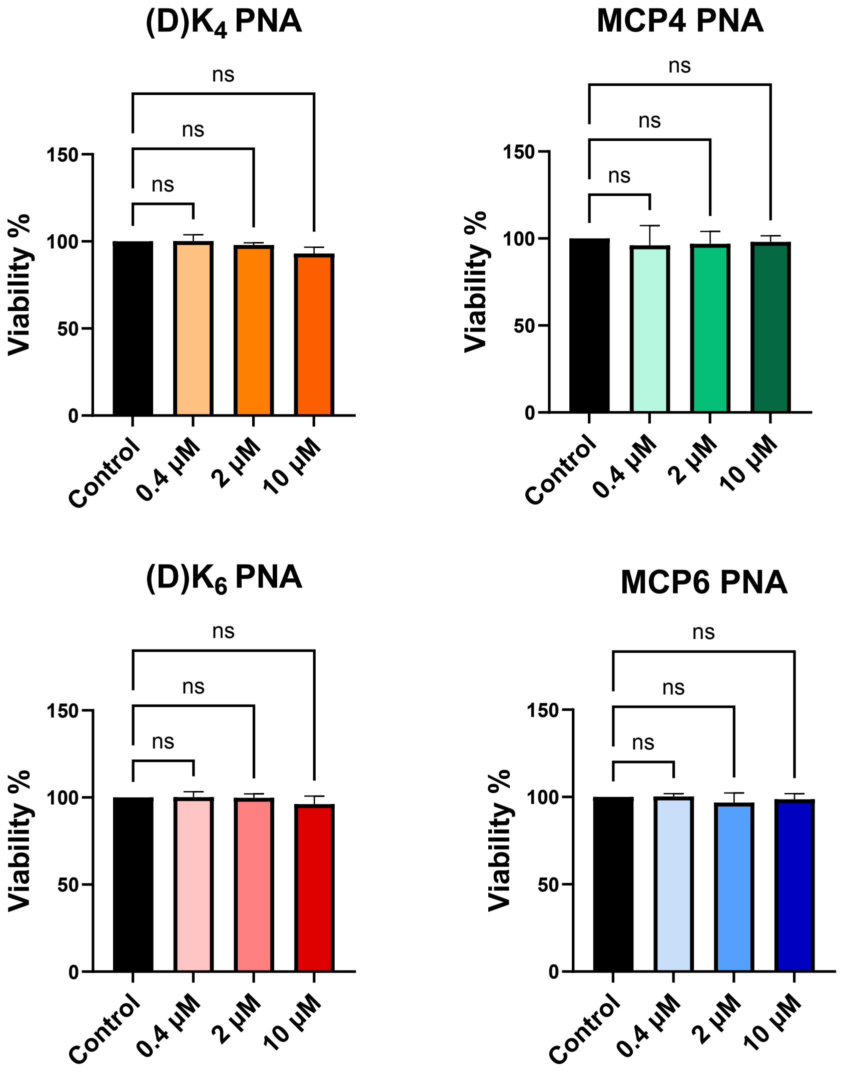

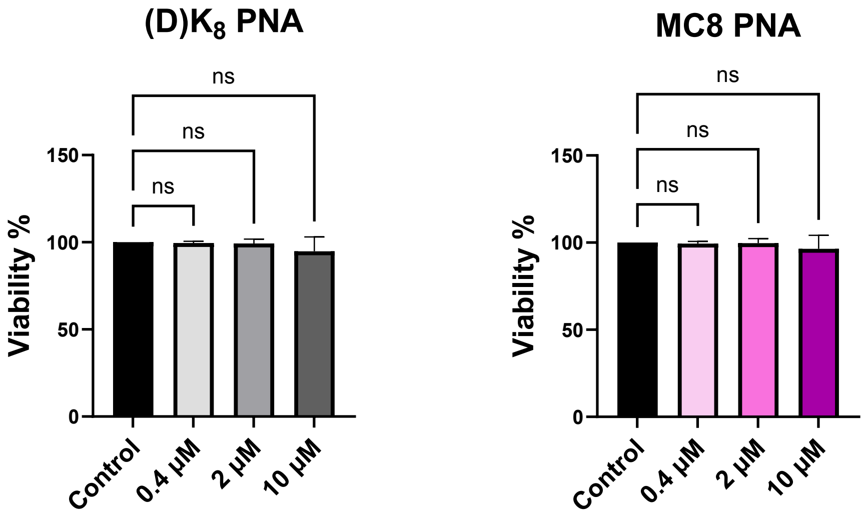

Figure S27: Viability of OVCAR-8 cells after 48 hours assessed by Crystal Violet staining. OVCAR-8 cells were treated with PNAs (0.4,2 and 10 µm) and incubated at 37 °C for 48 hours. The Data is presented as the mean ± SD (n = 3). *** represents $p \leq 0.001$, ** represents $p \leq 0.01$ and * represents $p \leq 0.05$ as determined by a One-way ANOVA test.

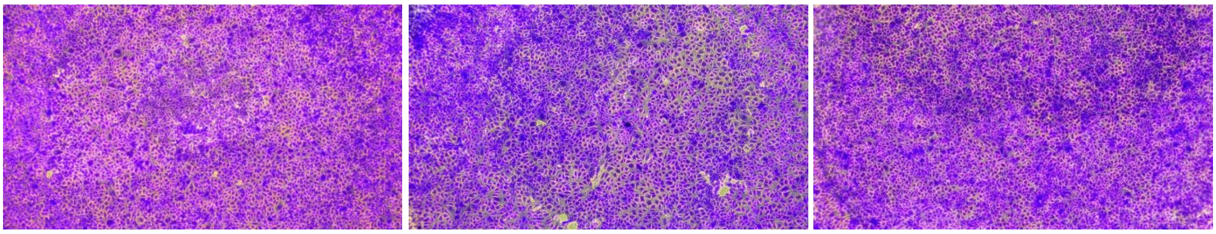

A) Control

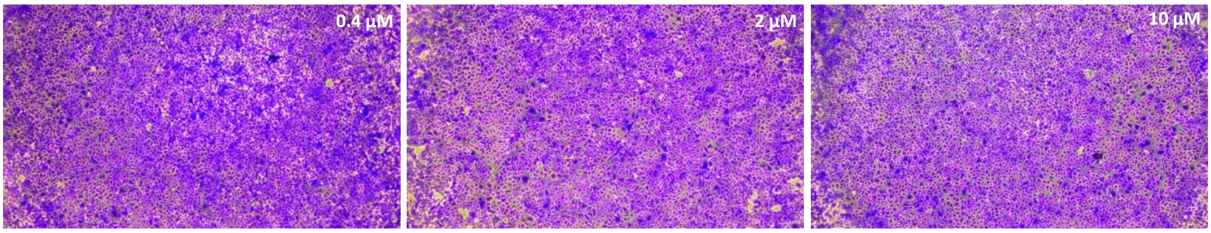

B) (D)K_4 PNA

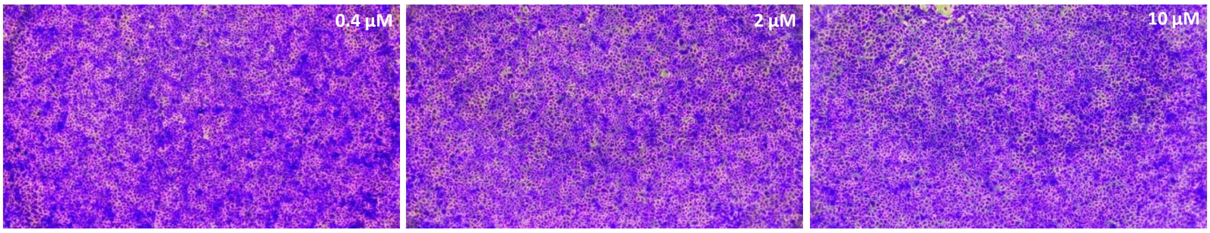

C) MCP4

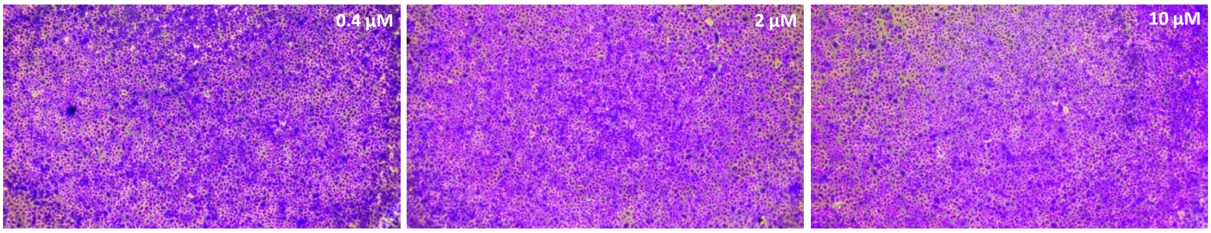

D) (D)K_6 PNA

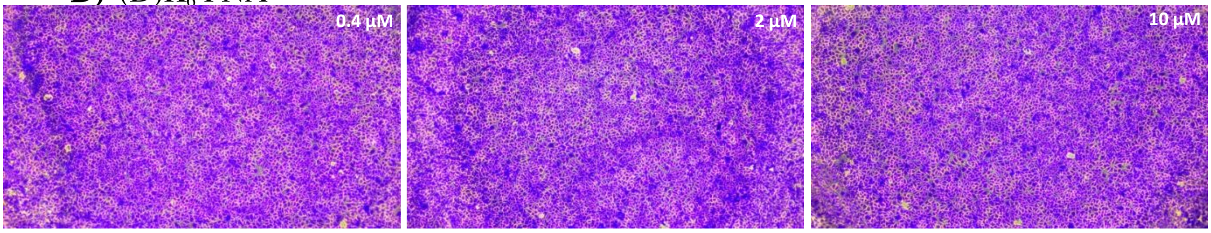

E) MCP6

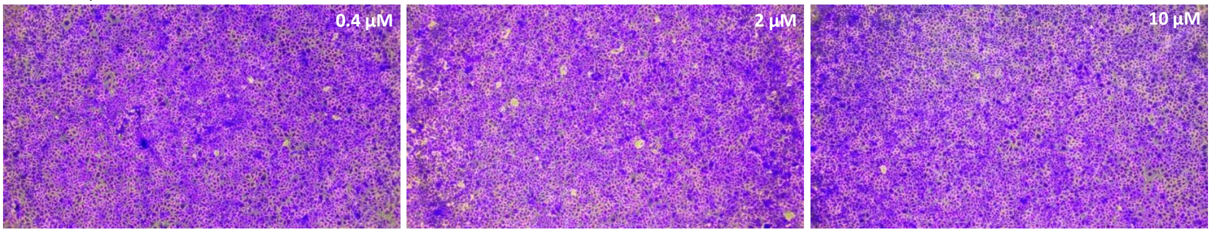

F) (D)K_8 PNA

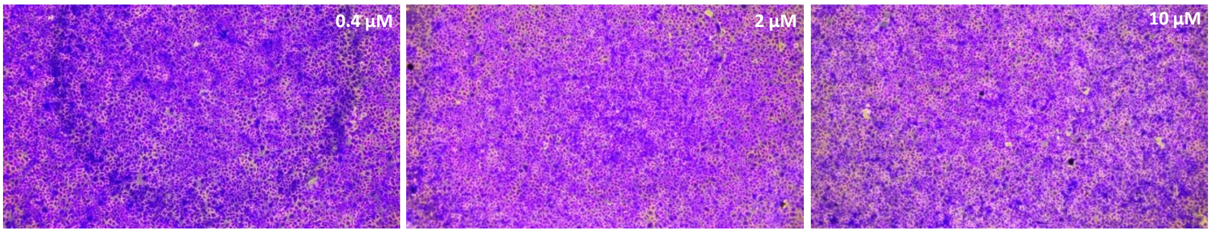

G) MCP8 PNA

Figure S28: Viability of OVCAR-8 cells after 48 hours assessed by Crystal Violet staining. OVCAR-8 cells were treated with PNAs (0.4,2 and 10 µm) and incubated at 37 °C for 48 hours. Untreated cells served as control. **(A)** Untreated OVCAR-8 cells (served as control), **(B)** (D)K_4 PNA, **(C)** MCP4 PNA, **(D)** (D)K_6 PNA, **(E)** MCP6 PNA, **(F)** (D)K_8 PNA and **(G)** MCP8 PNA.

Methylation of Fmoc-Bhoc-PNA(A)-OH

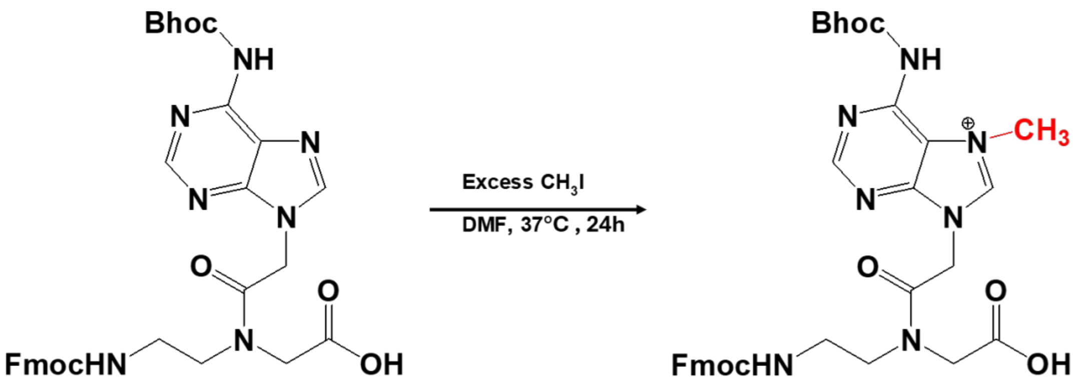

Scheme 1. Synthesis of A^+ monomer. Methyl group is marked in red.

A^+ PNA monomer was synthesized using the same protocol reported for G^+ PNA monomer with slight changes[1]: Fmoc-Bhoc-PNA(A)-OH (500 mg, 0.68 mmol) was dissolved in 5 mL of anhydrous DMF under an argon. To this solution, iodomethane (860 µL, 13.79 mmol, 20 equiv.) was added dropwise. The reaction mixture was stirred at 37 °C for 24 hours. Upon completion, the reaction was quenched by the addition of 100 mL of diethyl ether, and the mixture was stored at -20 °C overnight. The supernatant was carefully discarded, and 5 mL of water was added to the residue forming a yellowish precipitate. The crude product was collected by centrifugation and purified by HPLC. (Yield: 90 mg, 18%).

^{1}H and ^{13}C NMR spectra and HRMS of A^{+} PNA monomer

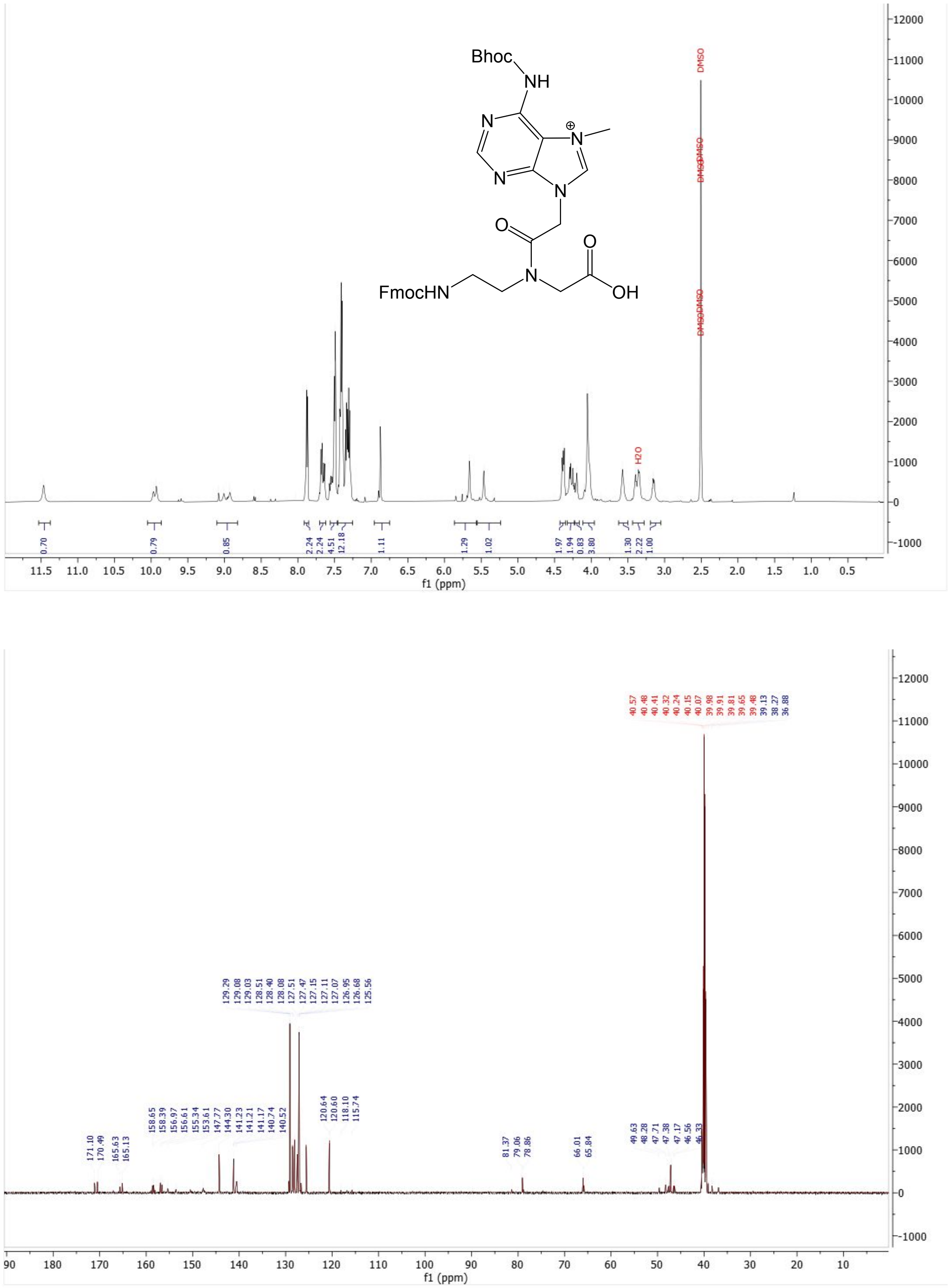

Figure S29: ^{1}H and ^{13}C NMR spectra of A^{+} PNA monomer.

^{1}H NMR (500 MHz, DMSO) δ 11.47 (s, 1H), 10.05 – 9.86 (m, 1H), 9.10 – 8.82 (m, 1H), 7.87 (d, J = 7.6 Hz, 2H), 7.66 (dd, J = 17.6, 7.6 Hz, 2H), 7.56 – 7.46 (m, 5H), 7.45 – 7.25 (m, 12H), 6.87 (s, 1H), 5.66 (s, 1H), 5.46 (s, 1H), 4.43 – 4.35 (m, 2H), 4.33 – 4.23 (m, 2H), 4.21 (d, J = 14.4 Hz, 1H), 4.04 (d, J = 14.2 Hz, 4H), 3.57 (t, J = 6.9 Hz, 1H), 3.44 – 3.28 (m, 2H), 3.15 (q, J = 6.6 Hz, 1H).

^{13}C NMR (126 MHz, DMSO) δ 171.10, 170.49, 165.63, 165.13, 158.65, 158.39, 158.12, 156.97, 156.61, 155.34, 153.61, 150.48, 147.77, 144.30, 141.23, 141.21, 141.17, 140.74, 140.52, 129.29, 129.08, 129.03, 128.51, 128.40, 128.08, 127.51, 127.47, 127.15, 127.11, 127.07, 126.95, 126.68, 125.56, 120.64, 120.60, 118.10, 115.74, 81.37, 79.06, 78.86, 66.01, 65.84, 49.63, 48.28, 47.71, 47.38, 47.17, 46.56, 46.33, 40.57, 40.48, 40.41, 40.32, 40.24, 40.15, 40.07, 39.98, 39.91, 39.81, 39.65, 39.48, 39.13, 38.27, 36.88.

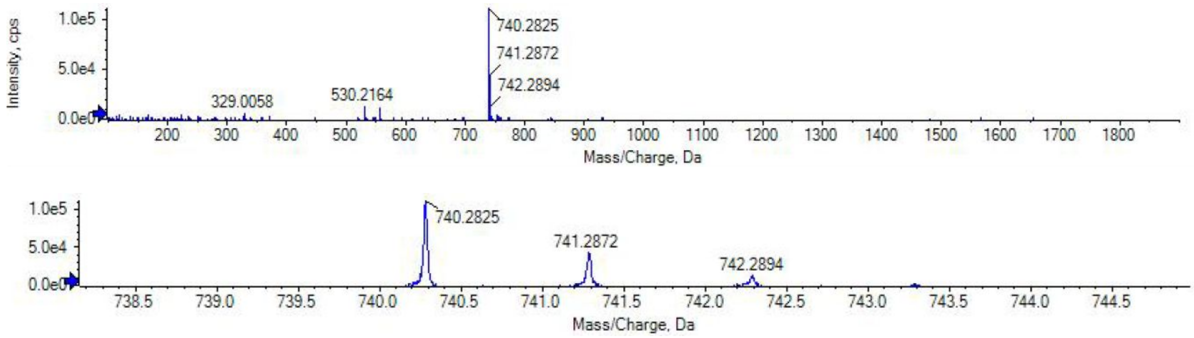

Figure S30: HRMS of A^+ PNA monomer. M_{calc} = 740.2827, M_{obs} =740.2825.

Reference:

1. M. Hibino, Y. Aiba and O. Shoji, Cationic guanine: positively charged nucleobase with improved DNA affinity inhibits self-duplex formation, *Chem Commun (Camb)*, 2020, **56**, 2546-2549.
